# SplinectomeR Enables Group Comparisons in Longitudinal Microbiome Studies

**DOI:** 10.3389/fmicb.2018.00785

**Published:** 2018-04-23

**Authors:** Robin R. Shields-Cutler, Gabe A. Al-Ghalith, Moran Yassour, Dan Knights

**Affiliations:** ^1^BioTechnology Institute, College of Biological Sciences, University of Minnesota, Minneapolis, MN, United States; ^2^Bioinformatics and Computational Biology, University of Minnesota, Minneapolis, MN, United States; ^3^Broad Institute of Massachusetts Institute of Technology, Harvard University, Cambridge, MA, United States; ^4^Center for Computational and Integrative Biology, Massachusetts General Hospital and Harvard Medical School, Boston, MA, United States; ^5^Department of Computer Science and Engineering, University of Minnesota, Minneapolis, MN, United States

**Keywords:** bioinformatics, microbiome analysis, R packages, computational biology methods, permutation tests, longitudinal data analysis

## Abstract

Longitudinal, prospective studies often rely on multi-omics approaches, wherein various specimens are analyzed for genomic, metabolomic, and/or transcriptomic profiles. In practice, longitudinal studies in humans and other animals routinely suffer from subject dropout, irregular sampling, and biological variation that may not be normally distributed. As a result, testing hypotheses about observations over time can be statistically challenging without performing transformations and dramatic simplifications to the dataset, causing a loss of longitudinal power in the process. Here, we introduce splinectomeR, an R package that uses smoothing splines to summarize data for straightforward hypothesis testing in longitudinal studies. The package is open-source, and can be used interactively within R or run from the command line as a standalone tool. We present a novel in-depth analysis of a published large-scale microbiome study as an example of its utility in straightforward testing of key hypotheses. We expect that splinectomeR will be a useful tool for hypothesis testing in longitudinal microbiome studies.

## Introduction

Biological studies in humans are subject to significant variability and noise, often great enough to obscure all but the most dramatic differences. Longitudinal studies are powerful in these cases, allowing researchers to observe (and account for) both inter- and intra-individual variability, or measure changes in response to an intervention in real time (Gonzalez et al., 2012; Gerber, 2014). As the costs of DNA sequencing have decreased, microbiome researchers have a greater opportunity to perform such longitudinal studies. While longitudinal data with multiple timepoints always provide more information than single-timepoint data, the computational tools to analyze longitudinal microbiome studies with multiple timepoints per subject lag behind. A number of practical concerns often complicate analysis of longitudinal microbiome data: time points are usually not in sync or differ in number between subjects, longitudinal variation may not follow a normal distribution, and timeseries data may follow arbitrary curves, for example during the maturation of the infant microbiome. To overcome these challenges, researchers in many studies have collapsed samples across time points to average individuals’ signals or they have summarized first with multivariate approaches that condense the initial observations (e.g., David et al., 2014; Zhou et al., 2015; Yassour et al., 2016). These approaches have been sufficient to make important discoveries in published studies, but there may still be opportunities to gain statistical power by using additional information content and directionality of the temporal axis.

To address this gap in data analysis, we introduce splinectomeR for direct hypothesis testing of categorical variables in longitudinal studies. SplinectomeR’s implementation is straightforward and complements recently developed mixed-effects models that are used for discovering differentiating taxa (Chen and Li, 2016). At the core of the tests is the *loess* spline that uses weighted local polynomials to model data that may not follow any classical model or shape (as is common in real biological data) (Cleveland, 1979; Cleveland and Devlin, 1988). Null distributions are generated by permutation of the data, similar to methods implemented in multivariate tests such as PERMANOVA (Anderson, 2001). Lastly, its implementation as an R package makes it practical and easy to adopt by microbiome researchers.

splinectomeR contains three key functions: *permuspliner*, which tests for an overall significant difference between two groups’ summary splines over the longitudinal time course; *sliding_spliner*, which interpolates across the group spline and tests for significance between two groups at each interval to illuminate regions of time where significant differences exist; and *trendyspliner*, which tests for a significant non-zero overall trend in a single population over time. To demonstrate the utility of these tests, we performed additional testing of key hypotheses in a large published cohort of 37 infant microbiomes sampled over the first 3 years of life (Yassour et al., 2016). SplinectomeR is open-source and freely available for download and installation on GitHub at https://github.com/RRShieldsCutler/splinectomeR.

## Methods and Implementation

**FIGURE 1:**
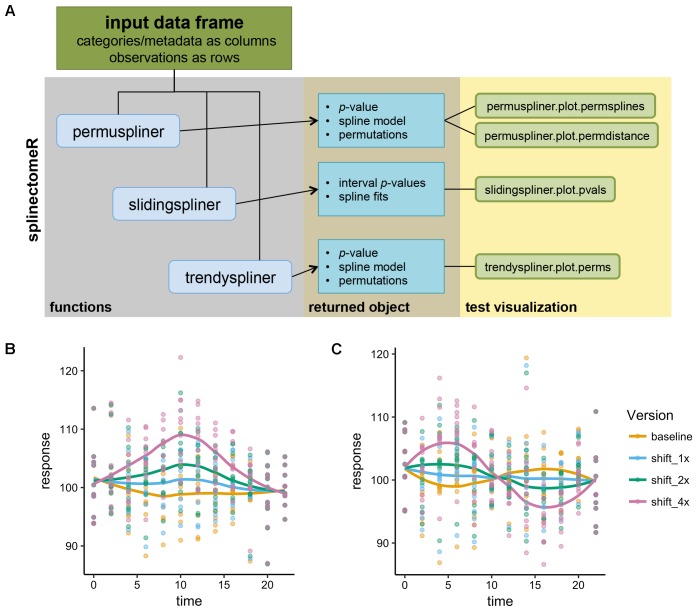
Schematic of the splinectomeR analysis package and simulated analyses. **(A)** Each of the three primary tests produces a results list object containing several data articles including the *p*-value(s), and which can also be used to generate pre-configured data visualizations. To demonstrate that the splinectomeR package detects non-linear changes between groups during a time series, data were simulated for 10 individuals over time and perturbed at three magnitudes (1x, 2x, 4x). The perturbation was done at one **(B)** or two **(C)** regions of the time series The permuspliner test finds that these changes are less likely to be random as the magnitude increases, as would be expected. In **(B)**, *p* = 0.3, *p* = 0.005, and *p* = 0.001 for 1x, 2x, and 4x shifts, respectively; in **(C)** *p* = 0.79, *p* = 0.36, and *p* = 0.002 for 1x, 2x, and 4x shifts, respectively.

splinectomeR contains three primary functions that test specific hypotheses about longitudinal trends (see **Figure 1** for schematic diagram). Each function uses *loess* splines to smooth longitudinal data before performing the specific statistical test. The input is a properly formatted data frame: each quantitative measurement or metadata category is its own column, and each row is a separate observation. This is a common structure for bioinformatics metadata, including in microbiome analyses, and therefore tests may be performed with little or no reformatting required. The standalone command-line version of the scripts requires a tab-delimited file of the same structure. We refer the reader to the online package vignettes (included as Supplemental Data Files 1 and 2) for examples of proper input data and for manual data wrangling techniques.

### Permuspliner

The objective of the permuspliner function is to test whether two groups of individuals follow more different trajectories over time (or along any continuous axis) than would be expected by random chance. It compares two groups over time without collapsing the timepoints to a single averaged point. Since differences between groups may not be consistent over time, and responses may even invert relative to one another over time, collapsing could mask or nullify important differences between groups. This problem can be avoided by considering the entire time series in a statistical comparison. Permuspliner fits a spline to the average time series of each group of individuals, and then measures the absolute area between the splines to determine the observed group difference.

Specifically, the input data is first split into the two groups to be compared, A and B. In some studies, participants may only appear once or twice in the dataset; to filter out samples from low-prevalence participants, the user may set a threshold with “*cut_low = n.*” A *loess* spline is fit to each group’s total time series, if it also meets the minimum data sparseness threshold set by the “*cut_sparse = n*” parameter. Lastly, the direction of the null hypothesis can be set with “*test_direction = [more/less]*”; this enables the user to test for not only group differences that are greater than expected by chance (*test_direction = “more,”* the default) but also differences smaller than expected by chance (*test_direction = “less”*). To calculate the observed group distance, points are interpolated along each spline and the sum of the areas across these points yields the group distance. Splinectomer uses 1000 points by default but this can be edited in the function arguments (with “*ints = n*”) to account for a longer time series or greater/lower resolution. This area determination is then recalculated after permuting the group assignments: to which group each participant belongs (A vs. B) is shuffled randomly without replacement. Permuting the group labels, as opposed to the participant data points, preserves the underlying distributions and patterns of the individuals’ timeseries curves. The permutation is repeated (default setting is 999 permutations) to generate a null distribution over the random between-group distances, from which an empirical *p*-value is calculated and reported by comparison to the observed distance. Because the random distribution is built from the observed values, it inherently reflects the noise and variability of the dataset, and is therefore tailored to each study’s unique character. SplinectomeR also includes plotting functions for visualizing the permuted distribution.

### Sliding Spliner

As a complement to the permuspliner test, the sliding spliner function allows the user to test the data series at defined intervals and ask whether the two groups of interest are significantly different at a any point in time during the time series. This is often challenging in clinical datasets where sampling is not coordinated between individuals, so analysis requires artificial binning or averaging across time in order to compare enough data at any one time point. Here, each individual—as opposed to the whole group, as in the permuspliner test above—is summarized by a spline, thus filling gaps across their time series. Every interpolated interval can then be tested as a complete distribution of all participants without binning or unnecessarily dropping samples. Low-prevalence participants are again filtered out with the “*cut_low*” parameter. Since the splines are not extrapolated beyond the start and end of the individual’s time series, the start and end regions may become less dense as fewer subjects have early and/or late samples. To account for this, the “*test_density = n*” argument sets the minimum number of participants required in each group to perform and report a *p*-value (default = 3). The output from this function is a table containing a *p*-value for each time interval, summarized by the companion plotting function where intervals at which the groups significantly diverge are visualized, and the points are scaled according to the data density—larger circles on the plot mean more data were used to calculate the *p*-value at that interval than for smaller circles.

### Trendyspliner

While testing for association between a categorical variable and a longitudinal variable is straightforward using regressions and correlations, methods for quickly testing whether a response is increasing or decreasing over time are less established. The trendyspliner function tests whether a set of responses in one group changes in a non-zero direction over the time series (or other continuous independent axis). A spline is fit to the data and interpolated across the number of set intervals. Non-zero change is measured as the area between the group spline and a linear baseline that is established from the start point of the group spline. Thus, if the pattern of observations does not meaningfully increase or decrease over time, the spline will not diverge from the baseline and the areas will remain small. To generate the null distribution, the time series within each individual is then permuted, and the spline is recalculated along with the area to the permuted baseline. The permutation is repeatedly executed to generate a random distribution of areas from which the two-sided *p*-value is calculated by comparison to the observed value. In some biological measurements, individuals’ initial values may be variable (e.g., body weight, height). To normalize these differences and improve the ability to detect an increase or decrease in these situations, the user can elect to “*mean_center*” the observations before calculations, which shifts each individuals’ mean over time to the group mean from all individuals. As in the other modes, a plotting function allows the user to visualize the permuted splines in the context of the real data.

### User Customization

In each of the above functions, the user can alter several additional parameters for specific applications. These include the spline span parameter, a standard spline parameter that determines how large the local smoothing neighborhood is, and therefore the degree of smoothing; the number of permutations, which influences the sensitivity of the test and *p*-value, as more permutations will allow a lower *p*-value to be detected but will also increase run time (default is 999 permutations); and the number of intervals over which to divide the data (larger values also increase run time and memory but provide finer resolution and more precise comparisons). These arguments allow flexibility for more advanced users with particular needs and unique data shapes.

### Package Implementation

The splinectomeR code was written in R version 3.4.0, and the package was built in RStudio using devtools and roxygen2 to generate and populate the package documentation (Wickham and Chang, 2017; Wickham et al., 2017). SplinectomeR is open-source and freely available on GitHub at https://github.com/RRShieldsCutler/splinectomeR. The figures generated by the secondary plotting functions are ggplot2 objects and can be saved in most image formats at a size and resolution specified by the user (e.g., through base R drivers or the “ggsave” function) (Wickham, 2009).

## Example Analysis

To demonstrate how splinectomeR detects group changes over a time series, we first generated a simulated data set. The response variable was perturbed at one or two regions of the time series, and at three magnitudes of change (**Figures 1B,C**). A linear model is a poor fit to these data shapes, and testing for absolute change from beginning to end is not sensitive to the internal dynamics. However, splinectomeR uses the entire dataset to compare between the baseline and perturbed data. As the magnitude of change increases, the permuspliner test yields decreasing *p*-values as expected in both the single region (*p* = 0.3, *p* = 0.005, *p* = 0.001 for 1x, 2x, and 4x shifts, respectively; **Figure 1B**) and double region perturbations (*p* = 0.79, *p* = 0.36, *p* = 0.002 for 1x, 2x, and 4x shifts, respectively; **Figure 1C**). Non-linear changes of this sort during a time series may be of great biological interest, although their statistical significance is difficult to test with existing tools.

Freely available datasets were used to test and further demonstrate the splinectomeR functions, as documented in the package vignettes that are available to view online in HTML format at https://rrshieldscutler.github.io/splinectomeR/. A proof-of-concept analysis was performed on the ChickWeights dataset in the R “datasets” package, and is available as a vignette on the website. To demonstrate splinectomeR’s utility on a more complex dataset, we used the publicly available OTU tables and associated metadata from a published longitudinal study of infant microbiomes by Yassour et al. (2016).

### Analysis of Longitudinal Microbiome Data

We tested splinectomeR’s utility on the taxonomic and metadata profiles from Yassour et al. (2016), to evaluate whether we could statistically support patterns described by the authors and investigate novel hypotheses. A more extensive analysis including all code used for data formatting is available as an online vignette and as Supplemental Data File 2.

**FIGURE 2:**
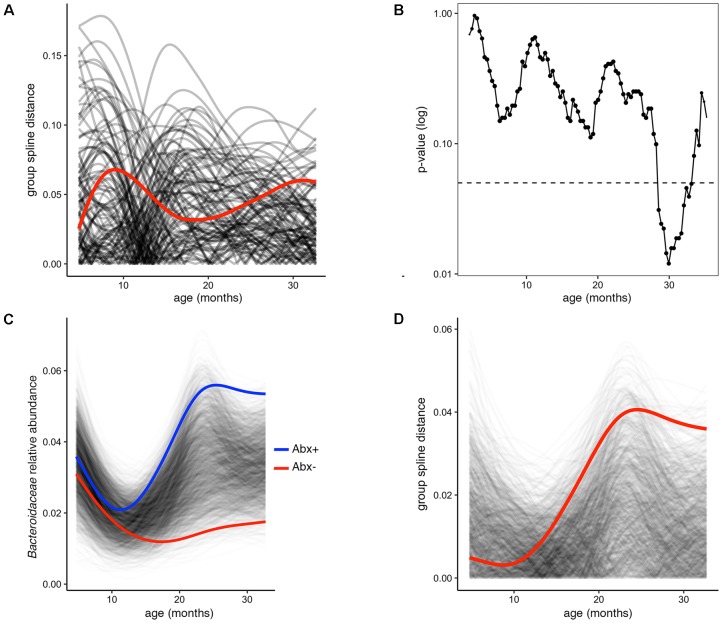
Permuted spline tests for statistical significance in longitudinal microbiome data. **(A)** The permuspliner output plot shows that the difference between *Bacteroidaceae* abundance between antibiotic exposed and non-exposed infants (distance between group splines shown as red line) is not significantly greater than 95% of the permuted values shown in translucent black. **(B)** The plot output of the sliding spliner function shows the *p*-value at each specified interval (shown with default 100 intervals) derived from the distribution of points from individuals’ smoothed splines. Dotted line indicates *p* = 0.05. The number of infants with data at a given interval is used to scale the data point size, as some entered and exited the study later or earlier, respectively. **(C)** *Porphyromonadaceae* abundance over time distinguishes antibiotic exposed (group spline in blue) and non-exposed infants (group spline in red; 999 permutations, *p* = 0.053). **(D)** Group distance plot, as in **(A)**, for the *Porphyromonadaceae* comparison, showing that permuted splines support a trend toward a greater true statistical difference after 12 months of life.

In several figures in the original publication, the authors draw visual comparisons between taxon abundances in antibiotic exposed versus non-exposed infants. These stream plots are powerful in displaying the inter-individual diversity and inspired us to use splinectomeR to perform statistical hypothesis testing on the time series data for differences between the infant groups. SplinectomeR’s permuspliner function can test whether a taxon’s abundance pattern over time is significantly different between antibiotic-exposed and non-exposed infants. We used splinectomeR to calculate that the difference in *Bacteroidaceae* abundance is not statistically significant across the overall time series (*p* = 0.28). The permutated differences support this conclusion, as the output shows in **Figure 2A**. However, the results indicate that the groups may be diverging toward the end of the time series, as the observed distance is higher relative to most of the permuted values. We tested this with the sliding spliner function, which generates a series of *p*-values across the longitudinal scale. The results, as the function’s output plot shows in **Figure 2B**, indeed show that there is a temporal pocket of significance surrounding the 30-month time point. We were also able to confirm the finding that the genus *Bacteroides* is significantly different between vaginal and cesarean born infants (*p* = 0.04), and that this difference is most pronounced in the first year of life (see Supplemental Data File 2).

Because these tests are implemented as R functions, they can be used programmatically for multiple hypothesis testing, such as testing all dominant bacterial families for significant differences between antibiotic exposure status. We performed this test on the present dataset (see vignette for full analysis), revealing that *Porphyromonadaceae* is the most discriminatory family (*p* = 0.05). The built-in plotting function (permuspliner.plot.permsplines) shows that antibiotic-exposed infants do indeed develop a notably higher abundance of *Porphyromonadaceae* (**Figure 2C**). The permuted splines lie predominantly between the two observed curves, supporting the conclusion that this difference is larger than expected by chance, and this observation becomes greater over time. The distance plot further supports this conclusion (**Figure 2D**), showing that initial distances are not greater than chance but become significant after approximately 12 months of age.

**FIGURE 3:**
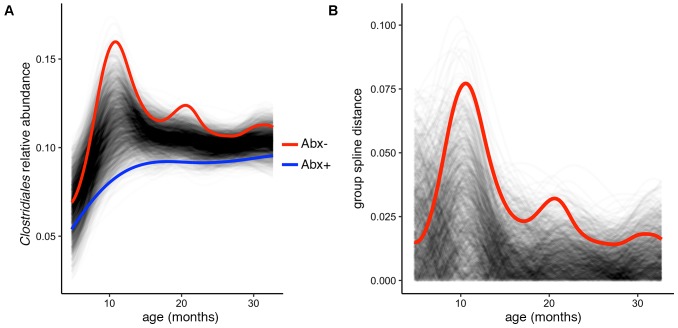
Complete time series analysis highlights a significant and temporally maintained deficiency in butyrate-producing *Clostridiales* among infants exposed to antibiotics. **(A)** Results plot generated by the permuspliner test, showing enriched abundance of *Clostridium* groups IV and XIV in infants not exposed to antibiotics (Abx–, red line). **(B)** Corresponding distance plot output, showing that the observed difference between the groups exceeds the random permuted distribution 997/1000 times, which supports the statistically significant finding (*p* = 0.003).

In their published analysis, the authors found a significant difference in abundance of the butyrate-producing *Clostridium* groups IV and XIV by antibiotic status at the final time point (36 months). Given butyrate’s important roles in gut homeostasis (e.g., Pryde et al., 2002; Ridaura et al., 2013; Zhang et al., 2016), it is worth investigating whether this difference exists over the infants’ first 3 years of development, or is just established at 36 months. To test this, we used the permuspliner function on a summarized table containing the following putatively butyrate-producing genera from *Clostridium* groups IV and XIV present in the OTU table: *Clostridium*, *Coprococcus*, *Dorea*, *Lachnospira*, *Roseburia*, *Ruminococcus*, and *Faecalibacterium* (Louis and Flint, 2009; Lopetuso et al., 2013; Van den Abbeele et al., 2013). When all of the longitudinal data are included in this comparison, we find that antibiotic exposed infants do indeed have significantly lower *Clostridium* group IV/XIV abundance compared to non-exposed infants over time (**Figure 3A**). Notably, our test using the entire 36-month time series yields a lower *p*-value than that reported using just the 36 month data: *p* = 0.003 vs. *p* = 0.037, respectively, and the resulting plot suggests that the most divergent time point is near 1 year of age (**Figures 3A,B**). This demonstrates the utility and simplicity of the splinectomeR tests; we are able to directly include 3 years of observations, strengthen support for this finding, and suggest directions for new hypotheses.

**FIGURE 4:**
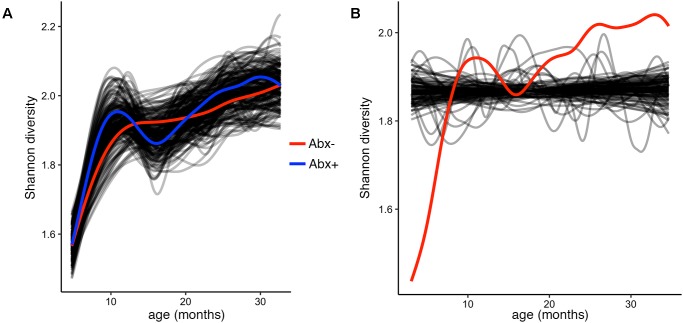
Alpha diversity increases over time but is not different between antibiotic exposure groups. (**A**) Output plot from the permuspliner test showing that Shannon diversity is not significantly different in infants exposed to antibiotics (Abx+, blue) compared to those who were not (Abx–, red), *p* = 0.96. **(B)** The results plot from the trendyspliner function shows that the permuted data form a zero-change distribution from which the real data (red line) is significantly distinct (*p* = 0.001). This supports the hypothesis that alpha diversity increases over time in the first 3 years of the infants’ lives.

To test and demonstrate the third function in the splinectomeR package, we hypothesized that the infants’ alpha diversity would significantly increase over time. We can use the permuspliner test to show that the alpha diversity is highly similar between the antibiotic (*p* = 0.92) and birth mode (*p* = 0.98) groups, but to statistically test whether it changes over time, we used the trendyspliner function. As shown in **Figure 4**, the randomly permuted splines generate a linear and zero-slope distribution, while the observed spline increases steadily over the time series, confirming our hypothesis that infant microbiome alpha diversity increases over time (*p* = 0.01). In this case, the trend is evident and expected; many biological datasets, however, have slight trends that are hard to interpret, in which case the trendyspliner test provides a straightforward permutation-based statistical test to confirm whether the deviation is greater than expected from chance.

### Summary

Yassour et al. (2016) present a prodigious dataset with dense longitudinal data that details the human gut microbiome’s complex dynamics over the first 3 years of life. The approaches presented above provide statistical support for observations and conclusions the authors reported, and allow us to test and develop additional hypotheses from the dataset. Researchers analyzing new longitudinal microbiome data with multiple samples per subject may benefit from these analytical capabilities provided by splinectomeR.

## Discussion

Longitudinal studies hold great promise for understanding the effects of interventions and environmental stimuli in the context of a naturally variable population. Analysis of these complex data has been impeded by a lack of clear, simple methods for directly comparing observations across multiple individuals and many time points without averaging or summarizing across time points. As we have demonstrated here using a large-scale longitudinal microbiome study, the splinectomeR package performs straightforward tests that are easy to interpret and will allow researchers to test important hypotheses from within R or a command line interface.

The approaches here are not without limitations; reliance on the *loess* spline means that the tests may be impacted by outliers, particularly in sparse datasets. User-definable arguments for sparseness cut offs and spline resolution (the smoothing parameter or spar) can help minimize these effects. Additionally, the size of longitudinal studies mean that the tests with many permutations can be slow to complete, though still easily performed on a standard workstation running R. From a standard metadata table, splinectomeR tests are run with a single command in R or on the command line. We provide the user with interpretable results in the form of pre-formatted plots that can be saved at publication quality and re-generated from the results object stored by the function.

## Conclusion

In summary, we have shown how a new open-source R package, splinectomeR, can quickly assess statistical significance in large longitudinal microbiome studies by summarizing longitudinal group data with splines and using randomly permuted distributions to evaluate the probability that the observed magnitude of differences between groups, or of trends over time, is due to chance. By providing three distinct types of hypothesis tests, we can explore overall changes in abundances or other biological measures, and compare longitudinal trends between groups of interest. Altogether, we are able to perform these tests in a way that takes full advantage of longitudinal data and maintains individual observations, thus leveraging all possible data points. Longitudinal studies generating “big data” with multi-omics approaches are now commonplace, but we lack appropriate tools to interpret these data. We offer splinectomeR as an open-source solution to testing key hypotheses in complex longitudinal data. SplinectomeR may also simplify analysis for longitudinal studies beyond the microbiome research field.

## Author Contributions

RS-C, GA-G, and DK conceived and designed the spline tests. RS-C and GA-G wrote the R code, and RS-C built the R package and conceived, and performed the example experiments. RS-C and MY analyzed the experiments. RS-C and DK wrote the manuscript.

## Conflict of Interest Statement

DK serves as CEO and holds equity in CoreBiome, a company involved in the commercialization of microbiome analysis. The University of Minnesota also has financial interests in CoreBiome under the terms of a license agreement with CoreBiome. These interests have been reviewed and managed by the University of Minnesota in accordance with its Conflict-of-Interest policies. The other authors declare that the research was conducted in the absence of any commercial or financial relationships that could be construed as a potential conflict of interest.
