# Supplementary material for: SplinectomeR Enables Group Comparisons in Longitudinal Microbiome Studies
Source: Front Microbiol. 2018 Apr 23;9:785. doi: 10.3389/fmicb.2018.00785 (PMC5924793; doi:10.3389/fmicb.2018.00785)
Supplement: Supplementary file 1 [file Data_Sheet_1.PDF]

Supplementary Material

SplinectomeR enables group comparisons in longitudinal microbiome studies

Robin R. Shields-Cutler, Gabriel A. Al-Ghalith, Moran Yassour, Dan Knights*

*** Correspondence:** Dan Knights: dknights@umn.edu

1 Supplementary Data

The two complete splinectomeR vignettes are included with this article as Supplemental Data in PDF format. Together, the vignettes demonstrate both data handling techniques and provide many examples of how the functions and plots in the splinectomeR package may be used.

Supplemental_Data_File_1: The ChickWeights vignette uses a built-in R dataset that is already in an appropriate format for testing with splinectomeR, and is presented in a tutorial format that users can follow along to learn about the package.

Supplemental_Data_File_2: The Yassour et al (2016) vignette provides a more "real-world" example of this package's utility.
