# Supplementary material for: SplinectomeR Enables Group Comparisons in Longitudinal Microbiome Studies
Source: Front Microbiol. 2018 Apr 23;9:785. doi: 10.3389/fmicb.2018.00785 (PMC5924793; doi:10.3389/fmicb.2018.00785)
Supplement: Supplementary file 2 [file Data_Sheet_2.PDF]

Analyzing Chick Weights with splinectomeR

Robin Shields-Cutler

2018-02-28

Contents

The ChickWeight Tutorial Vignette 1
Testing for differences between two diets 1
Sliding spline test 5
Non-zero trends 8
And indeed, that is what we see! 10

The ChickWeight Tutorial Vignette

Below you will find a simple splinectomeR tutorial/vignette based on the R dataset called `ChickWeight`. The purpose is to introduce new users to the package with a base R dataset, so anyone call follow along with their own R installation.
First, make sure that you have the datasets package and the splinectomeR package.

```
require(datasets)
require(ggplot2)
require(splinectomeR)

head(ChickWeight)  # Check out the structure of the ChickWeight data
```

```
##   weight Time Chick Diet
## 1     42    0     1    1
## 2     51    2     1    1
## 3     59    4     1    1
## 4     64    6     1    1
## 5     76    8     1    1
## 6     93   10     1    1
```

If you do not yet have splinectomeR installed, visit the GitHub page for installation options.
The ChickWeight dataset works well because it is a simple longitudinal study of several diets and their impact on chick body weights. With the `splinectomeR` package, we can test the following hypotheses: 1. Do the diets produce significantly different weight gain responses? 1. Are there significant differences along the time course? 1. Do all diets result in a non-zero weight gain (one would hope)?
Conveniently, the `ChickWeight` dataset is already in the ideal dataframe format for splinectomeR, where each row is an observation, and the diets, chick IDs, and weights are defined in individual columns.
Check the names of the columns, since these will be used in the splinectomeR functions, using `colnames()`.

```
## [1] "weight" "Time"   "Chick"  "Diet"
```

So, in our tests, the `xvar` will be "Time", the `cases` will be the "Chick", the `yvar` will be "weight", and the `category` of interest is the "Diet". These have to match exactly when entered into the functions below.

Testing for differences between two diets

To test for a difference between two diets, use the `permuspliner()` function with the variables highlighted above.

```r
result <- permuspliner(data = ChickWeight, xvar = 'Time',
                       yvar = 'weight', cases = 'Chick', category = 'Diet')
```

```
## Error in permuspliner(data = ChickWeight, xvar = "Time", yvar = "weight", : More than two groups in c
```

Oh no! The error is telling us there are more than two groups in the category we selected (Diet). If there are only two groups, the function will detect those automatically; if there are more than two (even a single third entry), you must either subset your data frame or specify which groups you want to compare with the `groups = ...` as a character vector (it will coerce this vector to character regardless, so here, `c('2','3')` could also be written `c(2,3)`).

```r
# Default permutations is 999, but we will set to 99 to speed up the code
permu_result <- permuspliner(data = ChickWeight, xvar = 'Time', yvar = 'weight',
                             cases = 'Chick', category = 'Diet',
                             groups = c('2','3'), perms = 99)
```

```
##
## Groups detected: 2 and 3 .
##
## Now testing between variables 2 and 3 for a difference in the response labeled weight
##
## Scalpel please: performing permusplinectomy with 99 permutations...
##
## Area between groups successfully calculated, now spinning up permutations...
## ...permutations completed...
##
## p-value = 0.17
##
##
## To visualize your results, try the following command, where "data" is your results object:
## permuspliner.plot.permdistance(data, xvar="Time")
## or
## permuspliner.plot.permsplines(data, xvar="Time", yvar="weight")
```

```r
# If you were to enter the 1,2 without quotes, it would return an error
# since it interprets groups as character strings not numbers.
```

So in this case, there is not a signficant difference between the weight changes of chicks on diet 1 vs diet 2. Let's try another.

```r
permu_result <- permuspliner(data = ChickWeight, xvar = 'Time', yvar = 'weight',
                             cases = 'Chick', category = 'Diet',
                             groups = c('1','4'), perms = 99)
```

```
##
## Groups detected: 1 and 4 .
##
## Now testing between variables 1 and 4 for a difference in the response labeled weight
##
## Scalpel please: performing permusplinectomy with 99 permutations...
##
## Area between groups successfully calculated, now spinning up permutations...
## ...permutations completed...
##
## p-value = 0.01
##
```

```
##
## To visualize your results, try the following command, where "data" is your results object:
## permuspliner.plot.permdistance(data, xvar="Time")
## or
## permuspliner.plot.permsplines(data, xvar="Time", yvar="weight")
```

In this example, the number of permutations was set to 99 for speed, but for a more sensitive p-value, set `perms = 999` (the default) or more.

There's a significant result. This means that the overall distance between these two data is greater than expected by chance. This can be hard to conceptualize, so the function is designed to save important features from the results so you can visualize the distances being compared.

The default result contains the reported p-value, the interpolated splines (as (x,y) points) for each group, and the spline objects for each group. These may be useful for downstream investigation, but for quick visualization you may choose to use the built-in plotting functions. To enable this feature, run the above commands with `retain_perms = T`.

```
permu_result <- permuspliner(data = ChickWeight, xvar = 'Time', yvar = 'weight',
                             cases = 'Chick', category = 'Diet',
                             groups = c('1','4'), perms = 999, retain_perm = T, ints = 100)
```

```
##
## Groups detected: 1 and 4 .
##
## Now testing between variables 1 and 4 for a difference in the response labeled weight
##
## Scalpel please: performing permusplinectomy with 999 permutations...
##
## Area between groups successfully calculated, now spinning up permutations...
## ...permutations completed...
##
## p-value = 0.002
##
##
## To visualize your results, try the following command, where "data" is your results object:
## permuspliner.plot.permdistance(data, xvar="Time")
## or
## permuspliner.plot.permsplines(data, xvar="Time", yvar="weight")
```

```
# Plot the real distance between the groups on top of all the permuted distances.
# Shows how far from the random distribution the real data lies across the time series
permuspliner.plot.permdistance(data = permu_result, xlabel = 'Time')
```

```
## Loading required package: reshape2
```

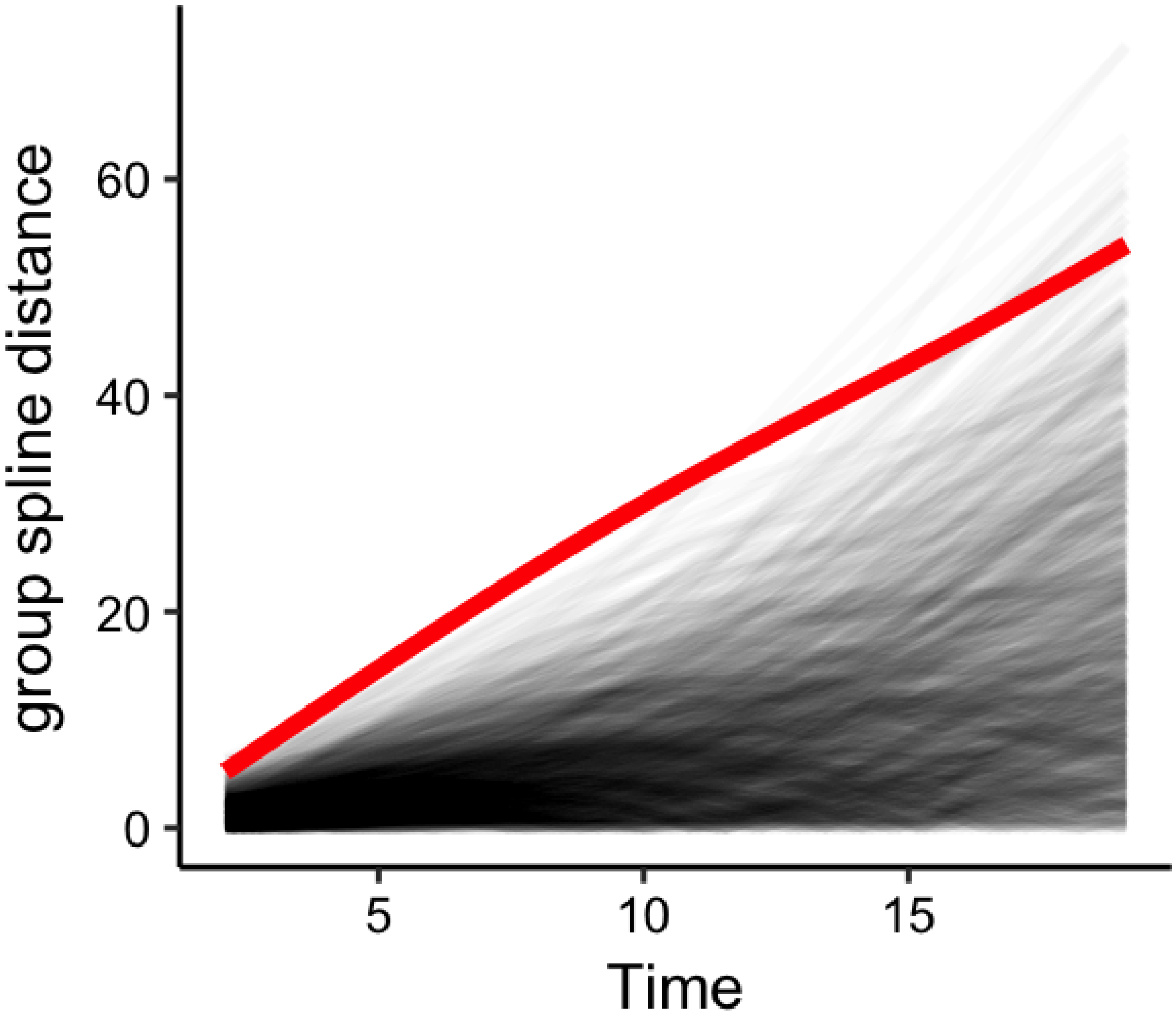


```
permuspliner.plot.permsplines(data = permu_result, xvar = 'Time', yvar = 'weight')
```

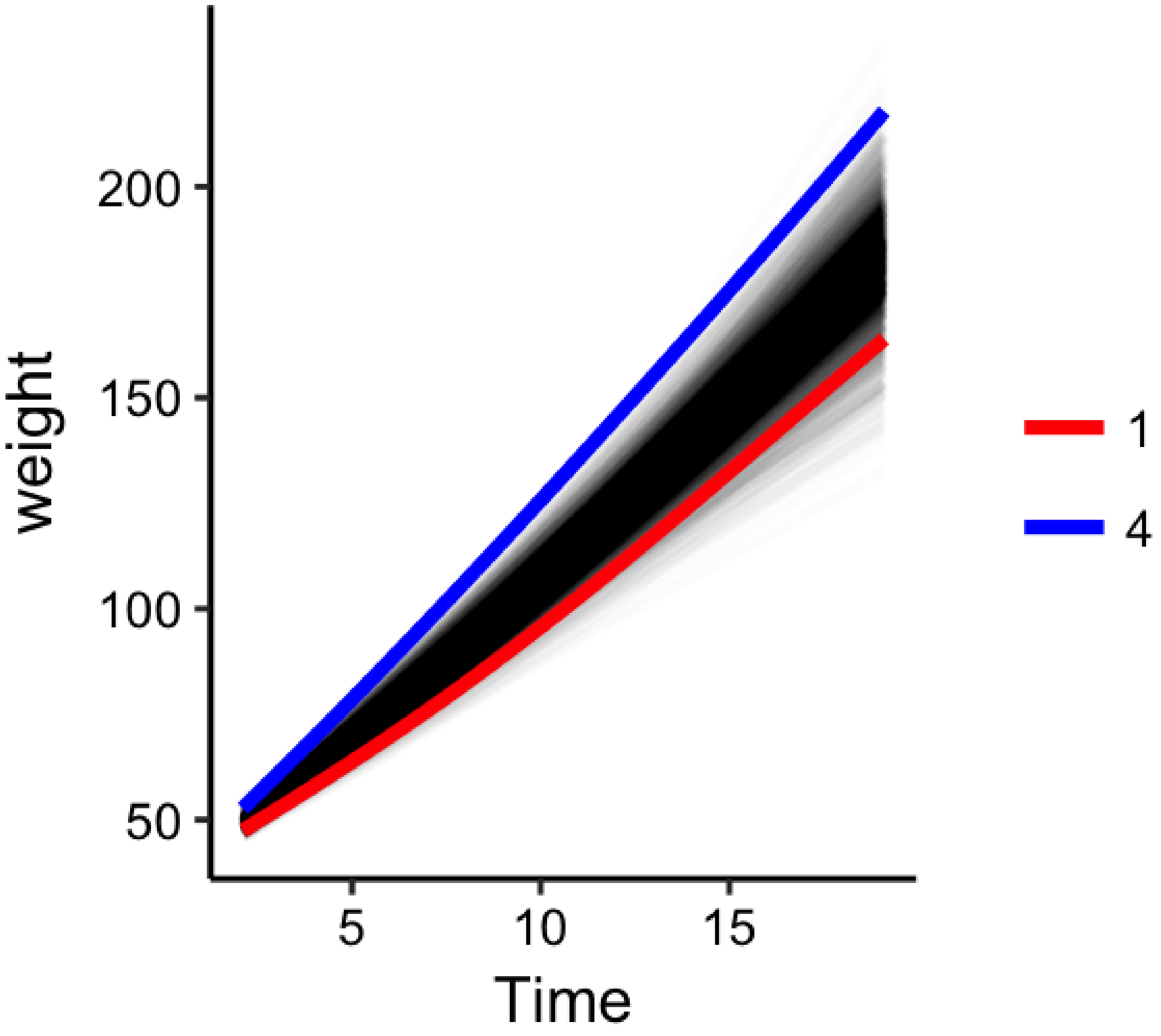

Sliding spline test

Let's say you are really interested in that diet 1 vs 2 comparison, even though it wasn't statistically significant overall. Perhaps there is a point in time where the weights are actually different, but it gets washed out over the whole timeseries.
To test this, you can use the `slidingspliner()` function. The result is a table of p-values, and can be quickly visualized using the built-in plotting function powered by `ggplot2`.

```
result <- sliding_spliner(data = ChickWeight, xvar = 'Time', yvar = 'weight',
                          cases = 'Chick', category = 'Diet', groups = c('1','2'))
```

```
## Running sliding spline test with 100 time points extrapolated from splines...
##
## Groups detected: 1 and 2 .
##
## Data organization successfull;
## ...now testing for significant differences in the response labeled weight
## Splines successfully generated for each case; now testing for significance over 100 intervals
## Testing completed, just organizing the results a bit...
```

```
##
## To visualize your results, try the following commands, where "data" is your results object:
## sliding_spliner.plot.splines(data, xvar="Time", yvar="weight", category="Diet")
## or
## sliding_spliner.plot.pvals(data, xvar="Time")
```

```r
sliding_spliner.plot.pvals(result, xvar = 'Time')
```

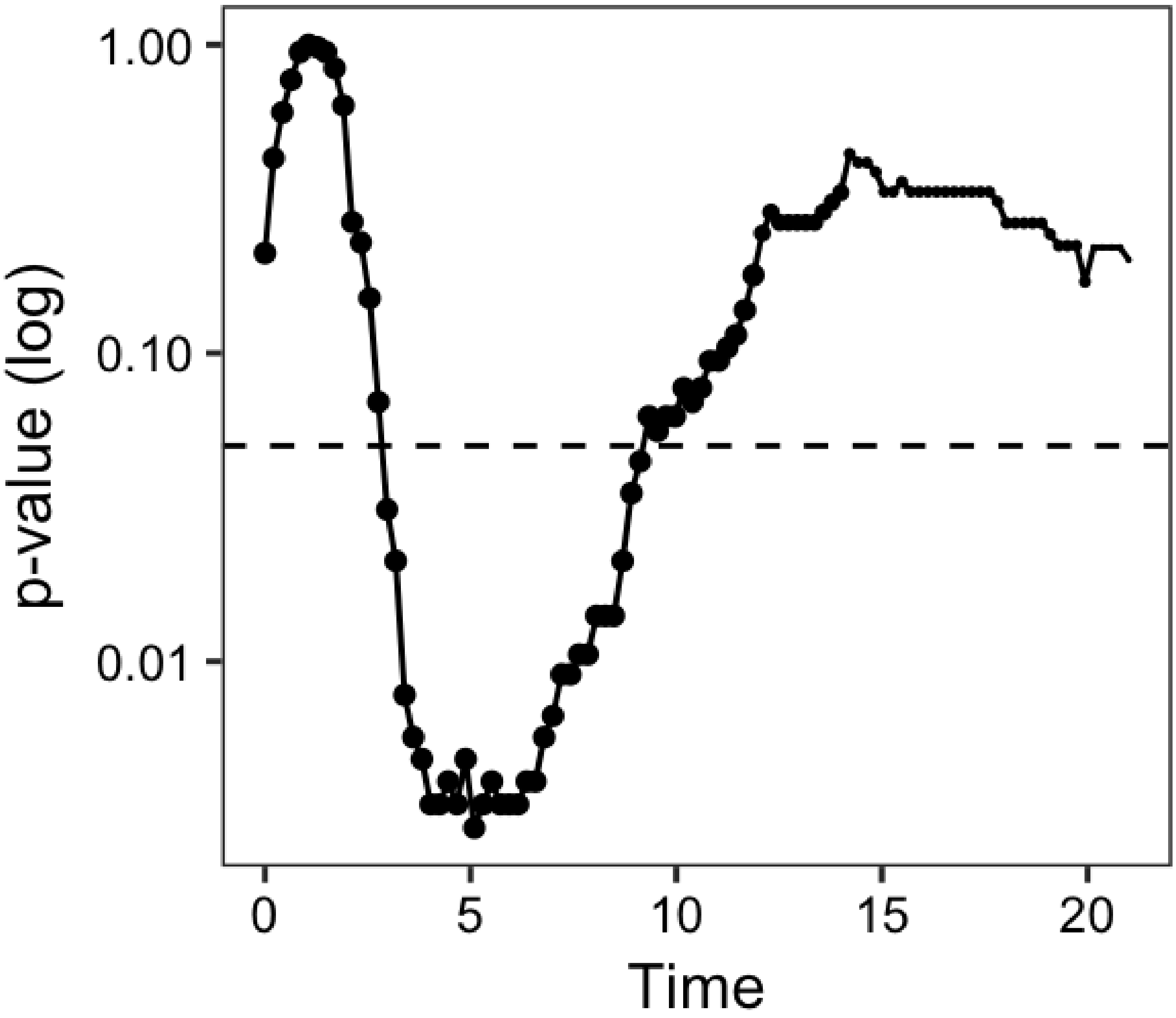

The defaul is 100 intervals, but if you have a very long or short timeseries, you might want more or less intervals, respectively. Here you can see what it looks like with just 5 intervals:

```r
result <- sliding_spliner(data = ChickWeight, xvar = 'Time', yvar = 'weight',
                          cases = 'Chick', category = 'Diet', groups = c('1','2'), ints = 5)
```

```
## Running sliding spline test with 5 time points extrapolated from splines...
##
## Groups detected: 1 and 2 .
##
## Data organization successfull;
## ...now testing for significant differences in the response labeled weight
```

```
## Splines successfully generated for each case; now testing for significance over 5 intervals
## Testing completed, just organizing the results a bit...
##
## To visualize your results, try the following commands, where "data" is your results object:
## sliding_spliner.plot.splines(data, xvar="Time", yvar="weight", category="Diet")
## or
## sliding_spliner.plot.pvals(data, xvar="Time")
```

```r
sliding_spliner.plot.pvals(result, xvar = 'Time')
```

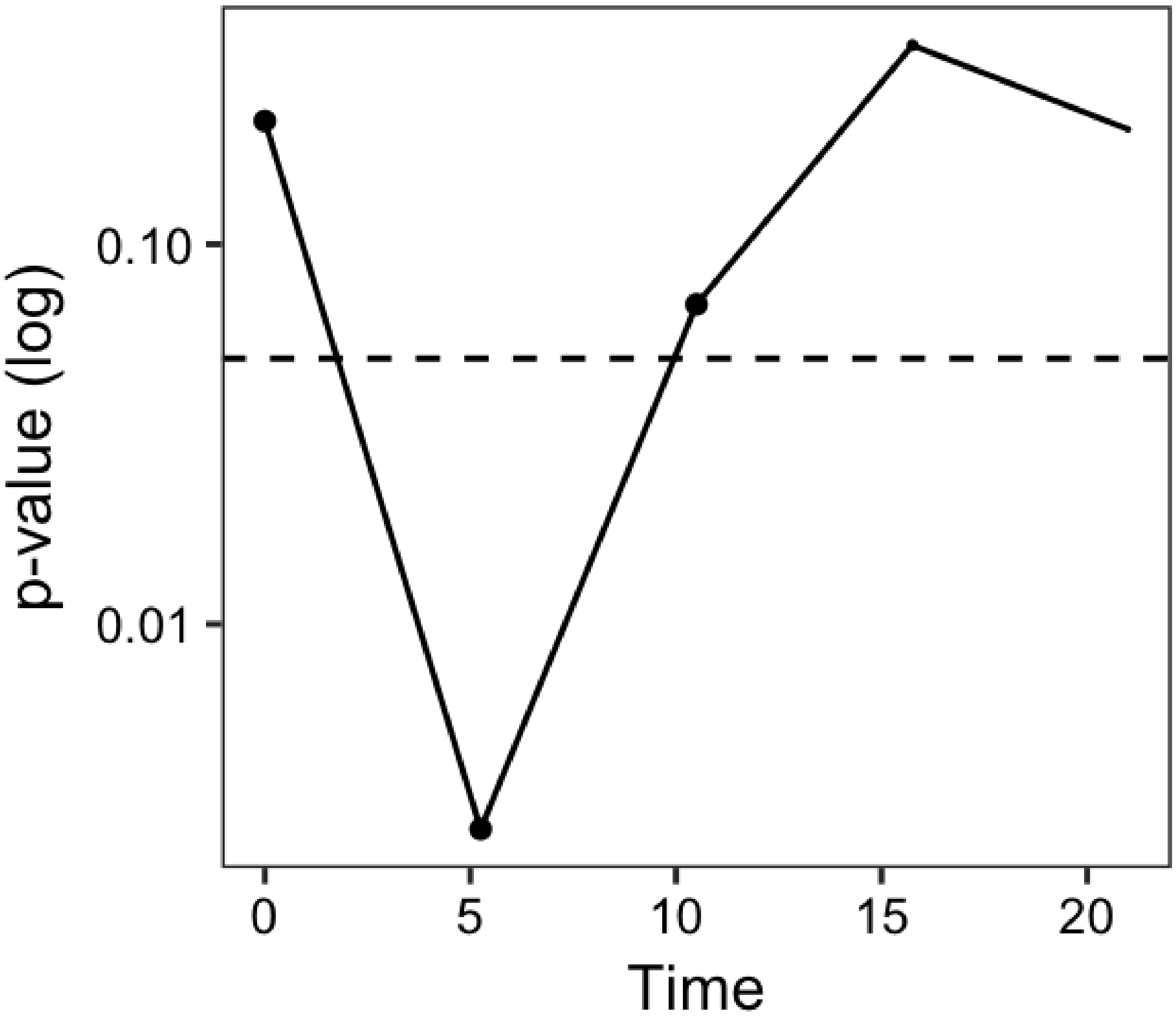

There is a line at $p = 0.05$, and the y-axis is log-scaled for visual clarity. You can always make your own plot from the stored results table as well, if this plotting does not fulfill your needs. Here, dots are scaled in size according to the number of original observations that support that interval; that is, the dots become smaller toward the 20 day time point because there are less overall data points in the dataset being used to calculate the splines and resulting p-values there. You can think of it as a quick visual confidence measure.

Non-zero trends

The third major functionality in the splinectomeR package is detecting significant non-zero trends through a permutation test, where the null hypothesis is a trend that doesn't differ from the group mean. Here, all of the Chicks are growing, so it is reasonable to predict that all of the diets will produce a non-zero trend.

As in the permuspliner() test above, you must specify a group to analyze if there are multiple groups.

```
diet_4_trend <- trendyspliner(data = ChickWeight, xvar = 'Time', yvar = 'weight', group = '4',
                        cases = 'Chick', category = 'Diet', perms = 99, mean_center = F)
```

```
##
## Testing for a non zero trend in weight across Time ...
##
## Performing the trendyspline test with 99 permutations...
## Calculations completed on observed data.
##
##               Now preparing the 99 permutations - this may take some time...
## Permutations completed successfully!
##
## p-value = 0.01
```

```
# There is also a plotting function for visualizing the permutations in this function
# Note the x and y label arguments are purely aesthetic
trendyspliner.plot.perms(diet_4_trend, xlabel = 'time', ylabel = 'weight')
```

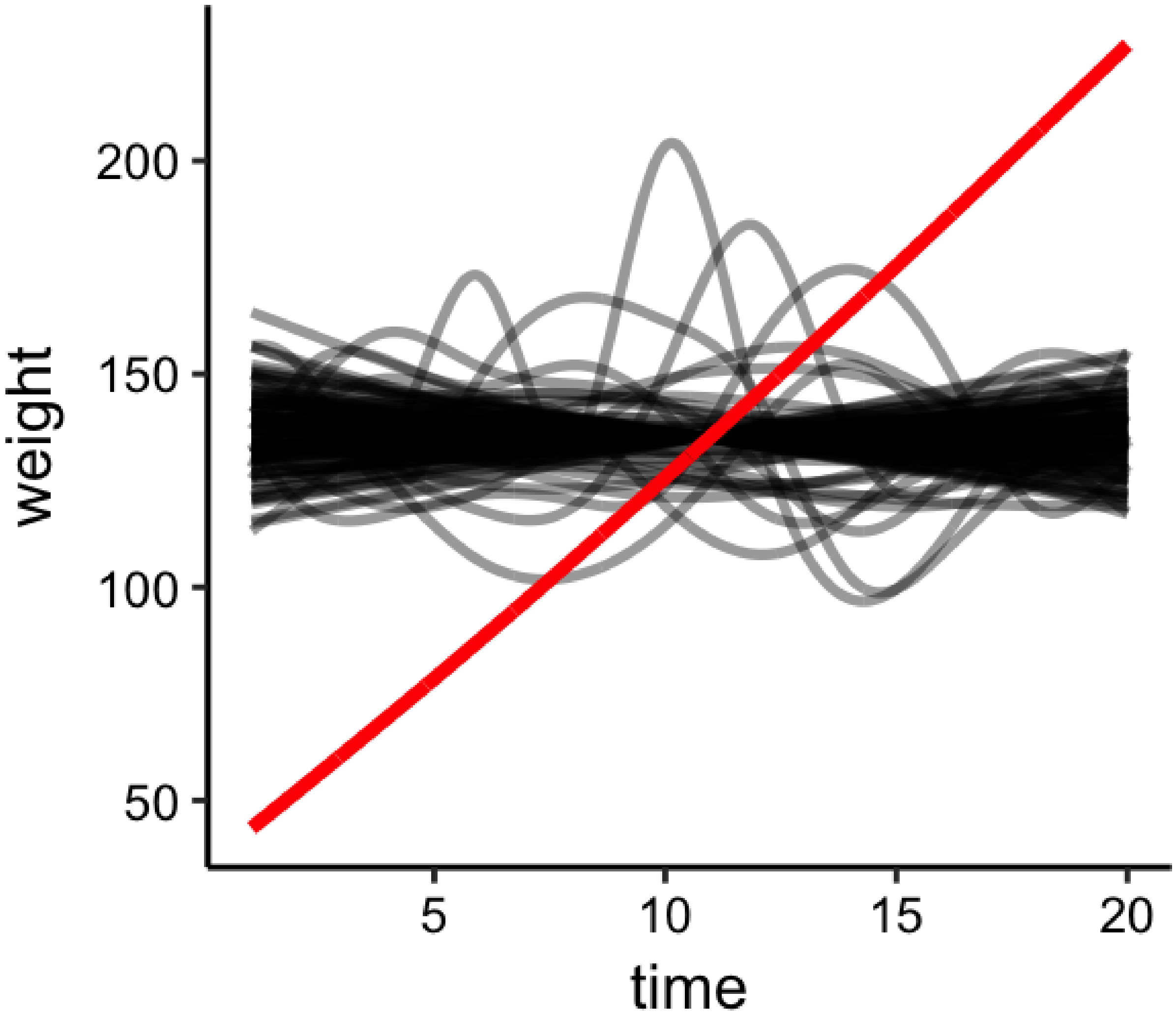

You could also quickly run through all four diet variables using some other R tricks (using `lapply()` here). We expect each of these diet conditions to have a positive weight trend, since in all cases the chicks are gaining weight:

```
diets <- c('1','2','3','4')
diet_results <- lapply(X = diets,
                       FUN = function(xx) trendyspliner(data = ChickWeight, xvar = 'Time',
                                                        yvar = 'weight', group = xx,
                                                        cases = 'Chick', category = 'Diet',
                                                        perms = 99, mean_center = F))
```

```
##
## Testing for a non zero trend in weight across Time ...
##
## Performing the trendyspline test with 99 permutations...
## Calculations completed on observed data.
##
##               Now preparing the 99 permutations - this may take some time...
## Permutations completed successfully!
##
```

```
## p-value = 0.01
##
##
## Testing for a non zero trend in weight across Time ...
##
## Performing the trendyspline test with 99 permutations...
## Calculations completed on observed data.
##
##               Now preparing the 99 permutations - this may take some time...
## Permutations completed successfully!
##
## p-value = 0.01
##
##
## Testing for a non zero trend in weight across Time ...
##
## Performing the trendyspline test with 99 permutations...
## Calculations completed on observed data.
##
##               Now preparing the 99 permutations - this may take some time...
## Permutations completed successfully!
##
## p-value = 0.01
##
##
## Testing for a non zero trend in weight across Time ...
##
## Performing the trendyspline test with 99 permutations...
## Calculations completed on observed data.
##
##               Now preparing the 99 permutations - this may take some time...
## Permutations completed successfully!
##
## p-value = 0.01
```

And indeed, that is what we see!

A couple notes and tips about this test: * This test is area-based so it is reasonalby tolerant to non-linear non-zero trends * In some data, the individual cases may have a range of starting points that could confound this trend. For example, measuring changes in many patients' blood glucose over time: some patients will start out higher than others, but it's the group trend (up or down vs linear/no change) that is of interest. In these situations you can mean-center the cases prior to analysis with the `mean_center = TRUE` argument. * If your data consists of only one group, you probably do not have a "category" column at all, and you can omit this arguement.
