# Supplementary material for: SplinectomeR Enables Group Comparisons in Longitudinal Microbiome Studies
Source: Front Microbiol. 2018 Apr 23;9:785. doi: 10.3389/fmicb.2018.00785 (PMC5924793; doi:10.3389/fmicb.2018.00785)
Supplement: Supplementary file 3 [file Data_Sheet_3.PDF]

Yassour et al (Science Trans Med, 2016) Antibiotics Cohort analysis with splinectomeR

Robin Shields-Cutler

2018-03-01

Contents

Demonstrating use of the splinectomeR package on a published dataset . . . 1
Data wrangling . . . 1
Data analysis . . . 4
Finally, applying the splinectomeR package . . . 5
Other hypotheses we can test . . . 20
Conclusions . . . 30

Demonstrating use of the splinectomeR package on a published dataset

This document shows both the data wrangling to generate easily splinectomeR-friendly dataframes from a standard taxa table, and the subsequent analysis demonstrating the splinectomeR functions.

The data here come from a study on the microbiome of Danish babies in the first 36 months of life. The picked OTU tables and corresponding metadata are available via the BROAD institute here, and their findings were published in the following article:
Yassour, et al. (2016) Natural history of the infant gut microbiome and impact of antibiotic treatment on bacterial strain diversity and stability. *Sci. Trans. Med.* 8(343)

The analysis below uses the 16S rRNA sequencing-based taxa table, included as package data here under the name `filtered_otu_table`. If you load the package with `library(splinectomeR)`, this data file should be accessible as an R object.

I saved each sheet of the metadata Excel files as a separate .txt file (though you could use an Excel-reading R package instead). I also discovered that there are a couple samples in the taxa table without metadata; similarly there is a sample in the metadata that doesn't have taxonomy data. I removed these from the metadata and any analysis below.

Data wrangling

```r
# First, load the required packages
library(dplyr)
library(tibble)
library(ggplot2)
library(reshape2)
library(tidyr)
library(splinectomeR)
library(vegan)
```

First, we can extract the samples and their corresponding timepoints from the taxa table's header.

```r
otus <- filtered_otu_table
sample_names <- colnames(otus)
sample_time <- lapply(sample_names, FUN = function(x) strsplit(x, split = '_'))
```

```r
timeseries <- data.frame(matrix(nrow = 1101, ncol = 2, data = 0))
j = 1
for (i in sample_time) {
  timeseries[j,1] <- i[[1]][1]
  timeseries[j,2] <- i[[1]][2]
  j = j + 1
}
colnames(timeseries) <- c('sample_id', 'month')
head(timeseries, n=4)
```

```
##   sample_id month
## 1   E022497  35.7
## 2   E023445  28.8
## 3   E010481  26.9
## 4   E000823  25.7
```

```r
# To save this table for reference:
# write.csv(timeseries, file = 'samples_timepoints2.csv', row.names = F, quote = F)
```

We can quickly then confirm the study IDs, and that we have the right longitudinal series by comparing these numbers to those in the paper. Next, we want to generate a full metadata/mapping file with information about whether each baby had antibiotics (Y/N), their breastfeeding status, delivery type, sex, and anything else. I combined the feeding rows with the general demographics in Excel before importing, and made the headers more R friendly (replaced spaces with underscores, etc). This data is included with the package, and is named `general_metadata`. The antibiotics sheet is likewise saved with the package as `antibiotics_metadata`.

```
##   #Sample_ID birth_year    sex       birth_mode gestational_age
## 1    E000823       2008   Male Vaginal delivery            39+4
## 2    E001958       2008 Female Vaginal delivery            40+1
## 3    E003188       2008 Female Vaginal delivery            38+6
## 4    E003953       2008 Female Vaginal delivery            39+5
##   months_final_exclusive_breastfeeding months_end_breastfeeding
## 1                                    5                    13.02
## 2                                    0                    12.66
## 3                                    5                    13.28
## 4                                    3                     6.98
##   months_started_formula_feeding feeding_in_hospital
## 1                             NA      OnlyBreastmilk
## 2                             NA               mixed
## 3                             10      OnlyBreastmilk
## 4                              3      OnlyBreastmilk
```

```r
# Create the binary antibiotic exposure annotation
abx <- antibiotics_metadata
abx_pos <- unique(abx$Subject)
abx_df <- data.frame(abx_pos, 'Y')
abx_neg <- setdiff(timeseries$sample_id, abx_pos)
abx_df2 <- setNames(data.frame(abx_neg, 'N'), c('abx_pos', 'X.Y.'))
abx_df <- rbind(abx_df, abx_df2)
colnames(abx_df) <- c('baby_id', 'antibiotics_y_n')
dim(abx_df)
```

```
## [1] 40  2
```

```r
head(abx_df, n=4)
```

```
##   baby_id antibiotics_y_n
```

```
## 1 E003188                 Y
## 2 E003953                 Y
## 3 E004628                 Y
## 4 E004898                 Y
```

```
# write.csv(abx_df, file = 'antibiotics_samplenames.csv', row.names = F, quote = F)
```

```
# Now, merge OTUs with each piece of the metadata
otus <- filtered_otu_table
otus <- data.frame(t(otus))
otus <- tibble::rownames_to_column(otus, var = 'sampleID')
otus_split <- otus %>% separate(sampleID, c('baby_id', 'timepoint'), sep = '_')
row.names(otus_split) <- otus$sampleID
otus_split <- rownames_to_column(otus_split, var = '#SampleID')

metadata <- general_metadata
colnames(metadata)[1] <- 'baby_id'
metadata <- merge(metadata, abx_df, by = 'baby_id')
# Merge the metadata with the OTU table to create a master metadata
# file including the taxonomy abundances
full_data <- merge(metadata, otus_split, by = 'baby_id')
x <- '#SampleID'
# Move the "#SampleID" column to the front:
full_data <- full_data[c(x, setdiff(names(full_data), x))]
# write_delim(full_data, 'full_taxa_metadata_yassour.txt', delim = '\t')
```

The paper focuses many of its analyses at the Family level of taxonomy. This next chunk parses out the family abundances from the OTU table.

```
otus <- filtered_otu_table
otus <- tibble::rownames_to_column(otus, var = 'OTU_ID')
otus_family <- otus %>% filter(!grepl('g__', otus$OTU_ID) & grepl('f__', otus$OTU_ID))
otus_family_sum <- otus_family
otus_family_sum$f__abun <- rowSums(otus_family[, 2:ncol(otus_family)])
otus_family_sum <- otus_family_sum[c('f__abun',
                                     setdiff(names(otus_family_sum), 'f__abun'))]
otus_family_sum <- otus_family_sum[order(-otus_family_sum$f__abun), ]
# write_delim(otus_family_sum, 'family_level_OTUs_with_sums.txt', delim = '\t')
# The top 10 Families in this dataset
otus_family_sum[1:10, 2]
```

```
##  [1] "k__Bacteria|p__Bacteroidetes|c__Bacteroidia|o__Bacteroidales|f__Bacteroidaceae"
##  [2] "k__Bacteria|p__Firmicutes|c__Clostridia|o__Clostridiales|f__Lachnospiraceae"
##  [3] "k__Bacteria|p__Firmicutes|c__Clostridia|o__Clostridiales|f__Ruminococcaceae"
##  [4] "k__Bacteria|p__Actinobacteria|c__Actinobacteria|o__Bifidobacteriales|f__Bifidobacteriaceae"
##  [5] "k__Bacteria|p__Proteobacteria|c__Gammaproteobacteria|o__Enterobacteriales|f__Enterobacteriacea
##  [6] "k__Bacteria|p__Proteobacteria|c__Betaproteobacteria|o__Burkholderiales|f__Alcaligenaceae"
##  [7] "k__Bacteria|p__Firmicutes|c__Clostridia|o__Clostridiales|f__Clostridiaceae"
##  [8] "k__Bacteria|p__Bacteroidetes|c__Bacteroidia|o__Bacteroidales|f__Porphyromonadaceae"
##  [9] "k__Bacteria|p__Firmicutes|c__Clostridia|o__Clostridiales|f__Veillonellaceae"
## [10] "k__Bacteria|p__Bacteroidetes|c__Bacteroidia|o__Bacteroidales|f__Rikenellaceae"
```

Check that the abundances roughly add up to 1 across the families in a sample:

```
colSums(otus_family_sum[, 3:10])
```

```
## E022497_35.7 E023445_28.8 E010481_26.9 E000823_25.7  E006781_8.7
```

```
##    0.9999998    0.9999992    1.0000006    0.9999996    1.0000006
##  E006781_8.0 E014403_15.2  E014403_4.2
##    1.0000003    1.0000012    0.9999994
```

Now, we're going to take the Family summarized data and merge that with the metadata. We'll be doing this same process several times during this analysis; this is a great opportunity to write a custom function that we can apply over and over.

```
# Define a function for all the tedious flipping, splitting, and merging
flip_split_merge <- function(otus_in, metadata) {
  row.names(otus_in) <- otus_in$OTU_ID
  otus_in$OTU_ID <- NULL
  otus_in <- data.frame(t(otus_in))
  otus_in <- tibble::rownames_to_column(otus_in, var = 'sampleID')
  otus_split <- otus_in %>% separate(sampleID, c('baby_id', 'timepoint'), sep = '_')
  otus_split$timepoint <- as.numeric(otus_split$timepoint)  # Trouble recognizing numbers
  row.names(otus_split) <- otus_in$sampleID
  otus_split <- rownames_to_column(otus_split, var = 'SampleID')
  otus_meta <- merge(metadata, otus_split, by = 'baby_id')
  otus_meta <- otus_meta[c('SampleID', setdiff(names(otus_meta), 'SampleID'))]
  return(otus_meta)
}
```

Now use the new function on the Family-summarized taxa table

```
family_tax_metadata <- flip_split_merge(otus_family, metadata)
# To save the table:
# write_delim(family_tax_metadata, 'family_taxa_metadata_yassour.txt', delim = '\t')
```

With this tool ready to go, we can now do some analysis from the data.

Data analysis

First, let's see if we can regenerate their first plot with the top ~6 bacterial Families (Fig. 1B). Before that, we'll make an ultra-longform table that will be absurdly long but easier to work with in wrangling and ggplot.

```
top_10_families <- otus_family_sum[1:10, 2]
top_10_families <- lapply(top_10_families, FUN = function(x) gsub(x, pattern = '|',
                                                                  replacement = '.',
                                                                  fixed = T))
top_family_meta <- family_tax_metadata %>%
  gather(key = 'family', value = 'relative_abundance',
         13:as.numeric(ncol(family_tax_metadata))) %>%
  filter(family %in% top_10_families)
# Double check that it worked:
unique(top_family_meta$family)
```

```
##  [1] "k__Bacteria.p__Actinobacteria.c__Actinobacteria.o__Bifidobacteriales.f__Bifidobacteriaceae"
##  [2] "k__Bacteria.p__Bacteroidetes.c__Bacteroidia.o__Bacteroidales.f__Bacteroidaceae"
##  [3] "k__Bacteria.p__Bacteroidetes.c__Bacteroidia.o__Bacteroidales.f__Porphyromonadaceae"
##  [4] "k__Bacteria.p__Bacteroidetes.c__Bacteroidia.o__Bacteroidales.f__Rikenellaceae"
##  [5] "k__Bacteria.p__Firmicutes.c__Clostridia.o__Clostridiales.f__Clostridiaceae"
##  [6] "k__Bacteria.p__Firmicutes.c__Clostridia.o__Clostridiales.f__Lachnospiraceae"
##  [7] "k__Bacteria.p__Firmicutes.c__Clostridia.o__Clostridiales.f__Ruminococcaceae"
##  [8] "k__Bacteria.p__Firmicutes.c__Clostridia.o__Clostridiales.f__Veillonellaceae"
##  [9] "k__Bacteria.p__Proteobacteria.c__Betaproteobacteria.o__Burkholderiales.f__Alcaligenaceae"
```

```
## [10] "k__Bacteria.p__Proteobacteria.c__Gammaproteobacteria.o__Enterobacteriales.f__Enterobacteriacea
```

Now we can plot! We're going to try to remake their Figure using ggplot2's `geom_smooth` function.

```
plot.df <- top_family_meta
plot.df <- separate(plot.df, col = family, sep = 'f__', remove = T,
                    into = c('uplevel', 'family'))
plot.df$uplevel <- NULL

ggplot(plot.df, aes(color = family, x = timepoint, y = relative_abundance)) +
  theme_bw() + geom_smooth(method='loess', span = 0.5) +
  labs(x = 'months', y = 'relative abundance') +
  theme(legend.position = 'right') + scale_x_continuous(breaks = c(0,12,24,36)) +
  theme(panel.grid = element_blank())
```

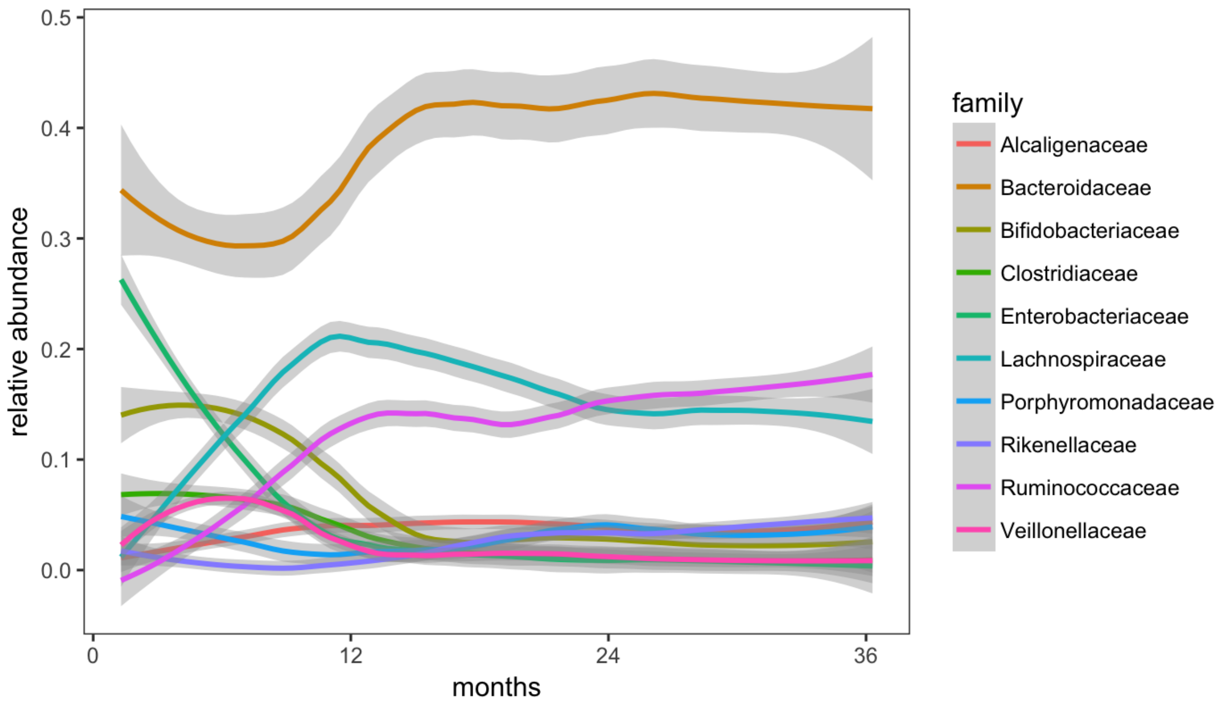

We included a few more families, but the plot is a very close match to Figure 1B.

Finally, applying the splinectomeR package

The `splinectomeR` package allows us to do a large number of statistical tests from this datatable using the longitudinal data. In the paper, statistical tests aren't performed across the intact longitudinal data, instead relying on visual comparisons of stream plots, for example, or on factors derived from the data, such as comparing consecutive-sample beta-diversity. With `splinectomeR`, we can quickly test whether abundance of a taxonomic unit (Family, Genus, etc) is significantly different across the time series between babies that had antibiotics and those who didn't, or male/female babies, birth mode, etc. We can also ask whether the abundance of a particular taxonomic unit is going up or down over time in a particular group (antibiotic exposed, female, etc).

Family level differences between categorical groups

```r
# Test the difference between Bacteroidaceae abundance in
#  antibiotic exposed vs. non-exposed conditions
f__Bacteroidaceae.top_family_meta <- top_family_meta %>% filter(family == 'k__Bacteria.p__Bacteroidetes
bacteroidaceae_result <- permuspliner(data = f__Bacteroidaceae.top_family_meta, xvar = 'timepoint',
                       yvar = 'relative_abundance', perms = 99,
                       category = 'antibiotics_y_n', cases = 'baby_id', retain_perm = T)
```

```
##
## Groups detected: N and Y .
##
## Now testing between variables N and Y for a difference in the response labeled relative_abundance
##
## Scalpel please: performing permusplinectomy with 99 permutations...
##
## Area between groups successfully calculated, now spinning up permutations...
## ...permutations completed...
##
## p-value = 0.28
##
## To visualize your results, try the following command, where "data" is your results object:
## permuspliner.plot.permdistance(data, xlabel="timepoint")
## or
## permuspliner.plot.permsplines(data, xvar="timepoint", yvar="relative_abundance")
```

```r
permuspliner.plot.permdistance(bacteroidaceae_result, xlabel = 'timepoint')
```

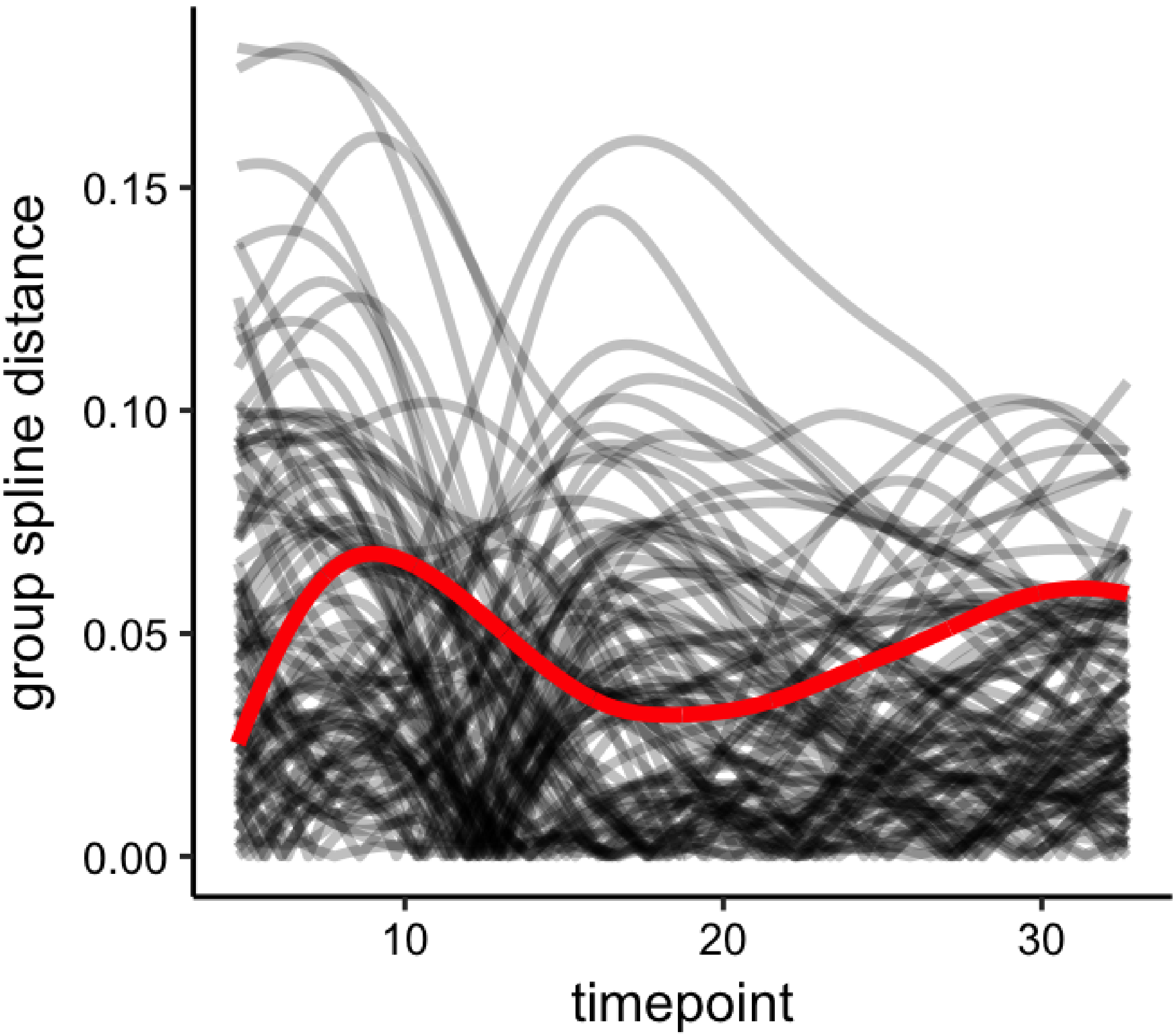

This tells us that over the time course, the difference in *Bacteroidaceae* abundance between antibiotic exposed and non-exposed infant microbiomes is not greater than expected by random chance. But, this is a very dynamic environment, and perhaps it is significant at some point along the time course. We can test that as well, using splinectomeR's `sliding_spliner` test.

```
slide_result <- sliding_spliner(data = f__Bacteroidaceae.top_family_meta,
                                xvar = 'timepoint', yvar = 'relative_abundance',
                                category = 'antibiotics_y_n', cases = 'baby_id',
                                test_density = 10, cut_low = 7,
                                set_spar = 0.5)
```

```
## Running sliding spline test with 100 time points extrapolated from splines...
##
## Groups detected: N and Y .
##
## Data organization successfull;
## ...now testing for significant differences in the response labeled relative_abundance
## Splines successfully generated for each case; now testing for significance over 100 intervals
## Testing completed, just organizing the results a bit...
```

```
##
## Done! To visualize your results, try the following commands, where "data" is your results object:
## sliding_spliner.plot.splines(data, xvar="timepoint", yvar="relative_abundance", category="antibiotics
## or
## sliding_spliner.plot.pvals(data, xvar="timepoint")
```

```
sliding_spliner.plot.pvals(data = slide_result, xvar = 'timepoint')
```

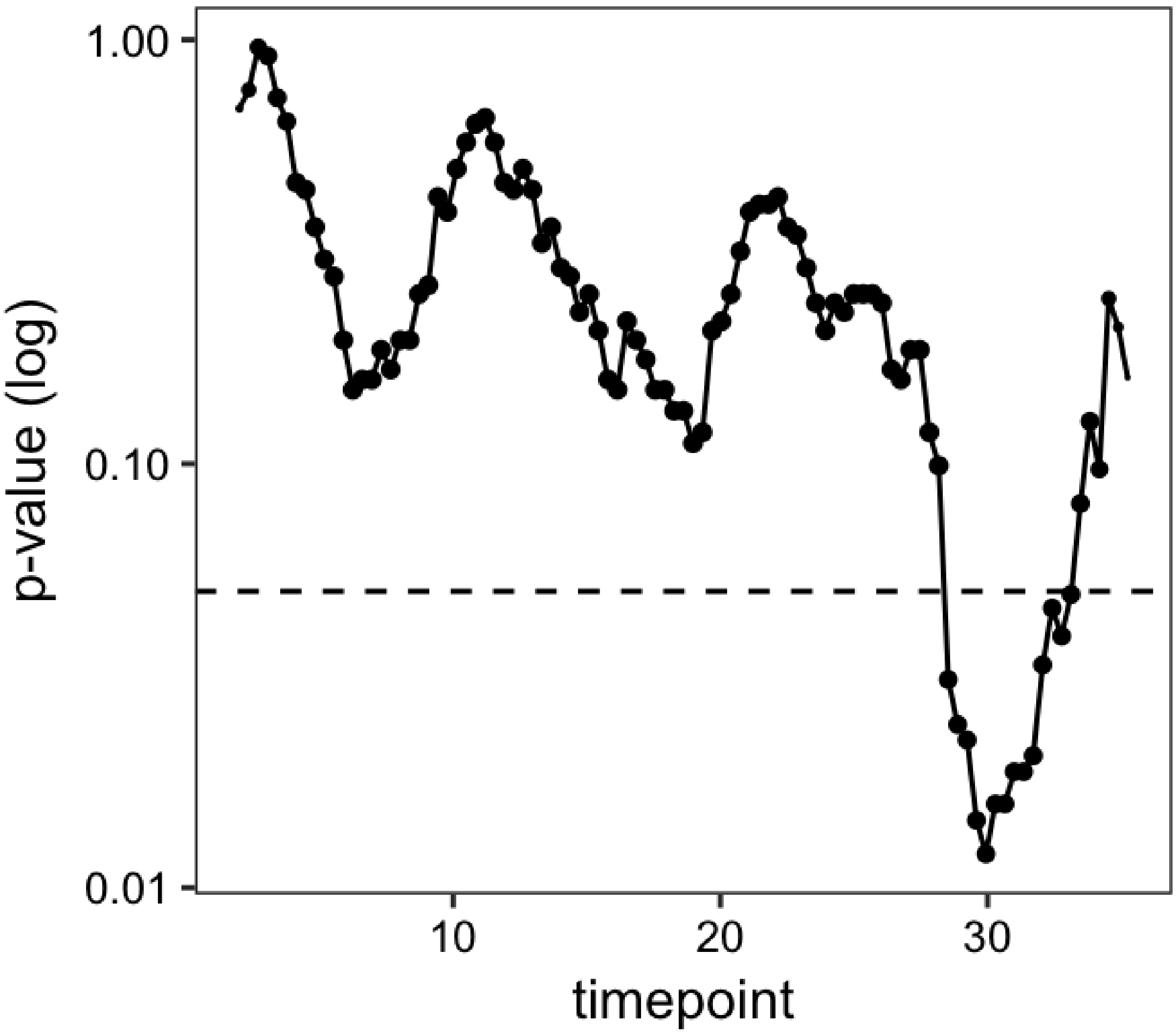

This shows that there are actually some significant differences near the 30-month window. There's also some interesting periodicity to the trend.

We might also ask whether *Bacteroidaceae* abundance increases or decreases overall as the babies get older. The `trendyspliner` test allows us to see if the overall change in *Bacteroidaceae* abundance is non-linear across all babies over the whole time course.

```
trendy_result <- trendyspliner(data = f__Bacteroidaceae.top_family_meta,
                               xvar = 'timepoint', quiet = F, cases = 'baby_id',
                               mean_center = T, yvar = 'relative_abundance', perms = 999)
```

```
##
```

```
## Testing for a non zero trend in relative_abundance across timepoint ...
##
## Performing the trendyspline test with 999 permutations...
##
## Ok, mean centering the data first... mean centering complete!
## Calculations completed on observed data.
##
##                Now preparing the 999 permutations - this may take some time...
## Permutations completed successfully!
##
## p-value = 0.001
```

We can use the same test and ask a similar question about other categorical variables. For exaple, maybe there are different trends in the 'mixed' vs 'only breastmilk' in-hospital feeding categories.

```
result_mixedfeed <- trendyspliner(data = f__Bacteroidaceae.top_family_meta,
                                  xvar = 'timepoint', quiet = T, cases = 'baby_id',
                                  mean_center = T, category = 'feeding_in_hospital',
                                  group = 'mixed', yvar = 'relative_abundance')
cat(result_mixedfeed$pval)
```

```
## 0.001
```

```
result_breastmilk <- trendyspliner(data = f__Bacteroidaceae.top_family_meta,
                                   xvar = 'timepoint', quiet = T, cases = 'baby_id',
                                   mean_center = T, category = 'feeding_in_hospital',
                                   group = 'OnlyBreastmilk', yvar = 'relative_abundance')
cat(result_breastmilk$pval)
```

```
## 0.001
```

Both are significant, meaning the abundance of *Bacteroidaceae* is non-linear over this time course. But remember, we'd have to plot it to know what direction each is going.

Automating over all the Families

Once we are analyzing in R, we can take a programmatic approach, and run the permutation test over all the top Families, and correct for false discovery rate on the resulting p-values.

```
top_20_families <- otus_family_sum[1:20, 2]
top_20_families <- lapply(top_20_families, FUN = function(x) gsub(x, pattern = '|',
                                                                   replacement = '.',
                                                                   fixed = T))
top20_family_meta <- family_tax_metadata %>%
  gather(key = 'family', value = 'relative_abundance',
         13:as.numeric(ncol(family_tax_metadata))) %>%
  filter(family %in% top_20_families)
# Double check that it worked:
unique(top20_family_meta$family)
```

```
##  [1] "k__Bacteria.p__Actinobacteria.c__Actinobacteria.o__Bifidobacteriales.f__Bifidobacteriaceae"
##  [2] "k__Bacteria.p__Bacteroidetes.c__Bacteroidia.o__Bacteroidales.f__Bacteroidaceae"
##  [3] "k__Bacteria.p__Bacteroidetes.c__Bacteroidia.o__Bacteroidales.f__Porphyromonadaceae"
##  [4] "k__Bacteria.p__Bacteroidetes.c__Bacteroidia.o__Bacteroidales.f__Prevotellaceae"
##  [5] "k__Bacteria.p__Bacteroidetes.c__Bacteroidia.o__Bacteroidales.f__Rikenellaceae"
##  [6] "k__Bacteria.p__Firmicutes.c__Bacilli.o__Lactobacillales.f__Lactobacillaceae"
##  [7] "k__Bacteria.p__Firmicutes.c__Bacilli.o__Lactobacillales.f__Streptococcaceae"
```

```
##  [8] "k__Bacteria.p__Firmicutes.c__Clostridia.o__Clostridiales.f__Clostridiaceae"
##  [9] "k__Bacteria.p__Firmicutes.c__Clostridia.o__Clostridiales.f__Lachnospiraceae"
## [10] "k__Bacteria.p__Firmicutes.c__Clostridia.o__Clostridiales.f__Ruminococcaceae"
## [11] "k__Bacteria.p__Firmicutes.c__Clostridia.o__Clostridiales.f__Veillonellaceae"
## [12] "k__Bacteria.p__Firmicutes.c__Clostridia.o__Clostridiales.f__unclassified"
## [13] "k__Bacteria.p__Firmicutes.c__Erysipelotrichi.o__Erysipelotrichales.f__Erysipelotrichaceae"
## [14] "k__Bacteria.p__Proteobacteria.c__Alphaproteobacteria.o__RF32.f__unclassified"
## [15] "k__Bacteria.p__Proteobacteria.c__Betaproteobacteria.o__Burkholderiales.f__Alcaligenaceae"
## [16] "k__Bacteria.p__Proteobacteria.c__Gammaproteobacteria.o__Enterobacteriales.f__Enterobacteriace
## [17] "k__Bacteria.p__Proteobacteria.c__Gammaproteobacteria.o__Pasteurellales.f__Pasteurellaceae"
## [18] "k__Bacteria.p__Verrucomicrobia.c__Verrucomicrobiae.o__Verrucomicrobiales.f__Verrucomicrobiacea
```

```
# Create a list containing each family's OTU+metadata table
f_top20_abx_pvals <- list()
for (f in unique(top20_family_meta$family)) {
  f__df <- top20_family_meta %>% filter(family == f)
  cat(f)
  f.result <- permuspliner(data = f__df, xvar = 'timepoint',
                       yvar = 'relative_abundance', perms = 99,
                       category = 'antibiotics_y_n', cases = 'baby_id',
                       cut_low = 6, quiet = T)
  cat(f.result$pval)
  f_top20_abx_pvals <- append(f_top20_abx_pvals, f.result$pval)
}
```

```
## k__Bacteria.p__Actinobacteria.c__Actinobacteria.o__Bifidobacteriales.f__Bifidobacteriaceae0.41k__Bac
```

```
f_top20_abx_qvals <- p.adjust(f_top20_abx_pvals, method = 'fdr')  # Adjusted p values
```

Let's see what the *Porphyromonadaceae* plot looks like. You could subset the table to this Family and plot with your favorite plotting package. Or, the `permuspliner` test includes two plotting functions. One plots the permuted splines along with the interpolated spline fit for the real data. The other one plots the distances between the two variables, and the distances between the permuted versions. In the former, a significant result would show most of the permuted mass lying between the real data splines.

```
Porphyromonadaceae.top_family_meta <- top20_family_meta %>%
  filter(family == 'k__Bacteria.p__Bacteroidetes.c__Bacteroidia.o__Bacteroidales.f__Porphyromonadaceae')
porph.result <- permuspliner(data = Porphyromonadaceae.top_family_meta,
                             xvar = 'timepoint',
                             yvar = 'relative_abundance',
                             category = 'antibiotics_y_n', cases = 'baby_id',
                             cut_low = 6, quiet = T, retain_perm = T)
cat(porph.result$pval)
```

```
## 0.053
```

```
permuspliner.plot.permsplines(data = porph.result,
                              xvar = 'timepoint',
                              yvar = 'relative_abundance')
```

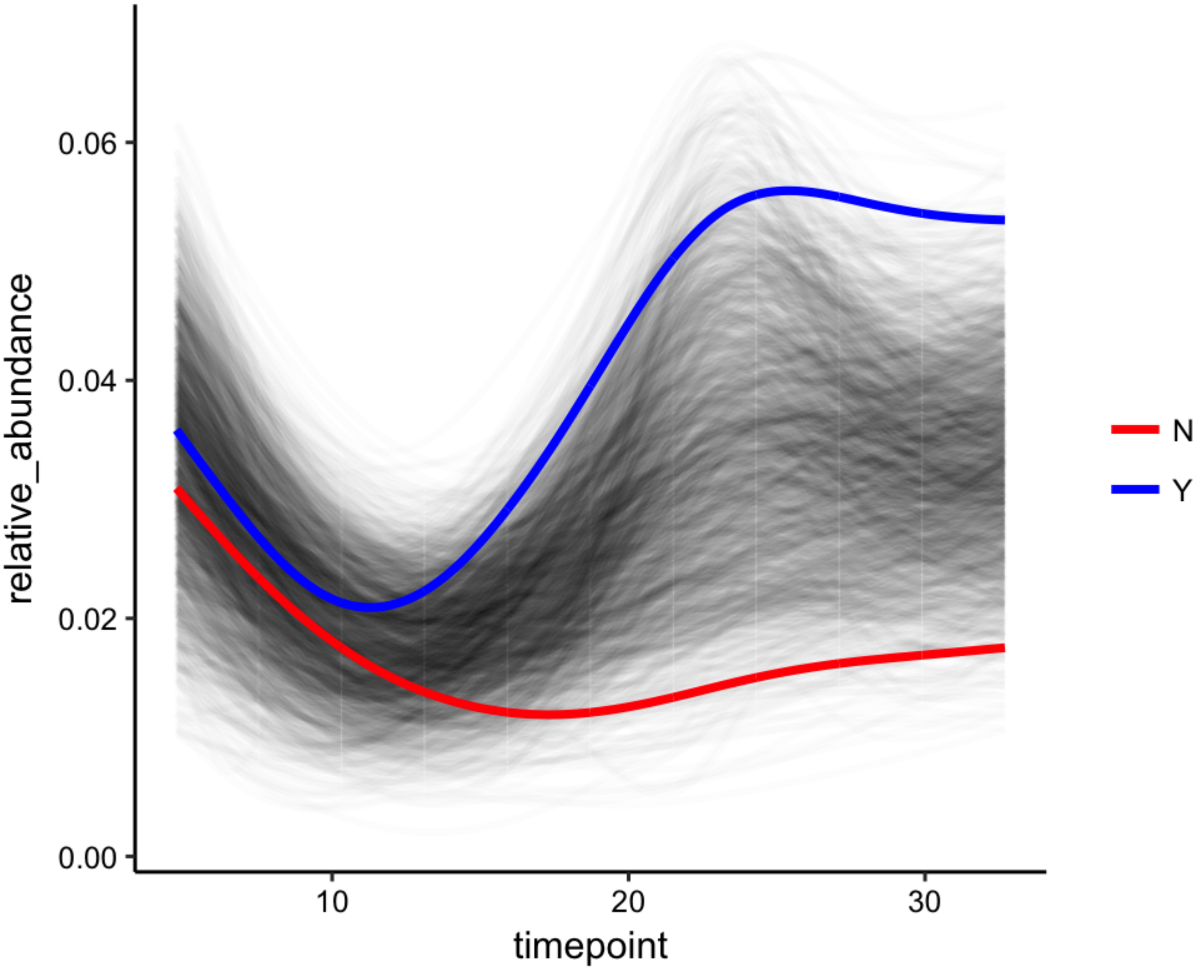

We can see from this plot that this taxon is becoming more discriminating between the groups over time. The translucent lines depict the permuted splines, which serves as a visual for how distinct the two groups are relative to random assortment. So, at the beginning, while the group splines are distinct, the permuted data covers a wide range above and below the observed data; this suggests that there is considerable noise and the difference is not strong here. In contrast, as time going on the two lines move farther apart and the permuted lines lay almost entirely between the real data; this indicates that the difference between these groups is stronger than you would see by random chance.

The second plotting function shows the distances between the two groups (N and Y antibiotics, here), directly visualizing the relationship between the observed difference and the random distribution of differences. So, a significant result here should show the bulk of permutation distances lying below the real data.

```
permuspliner.plot.permdistance(porph.result, xlabel = 'timepoint')
```

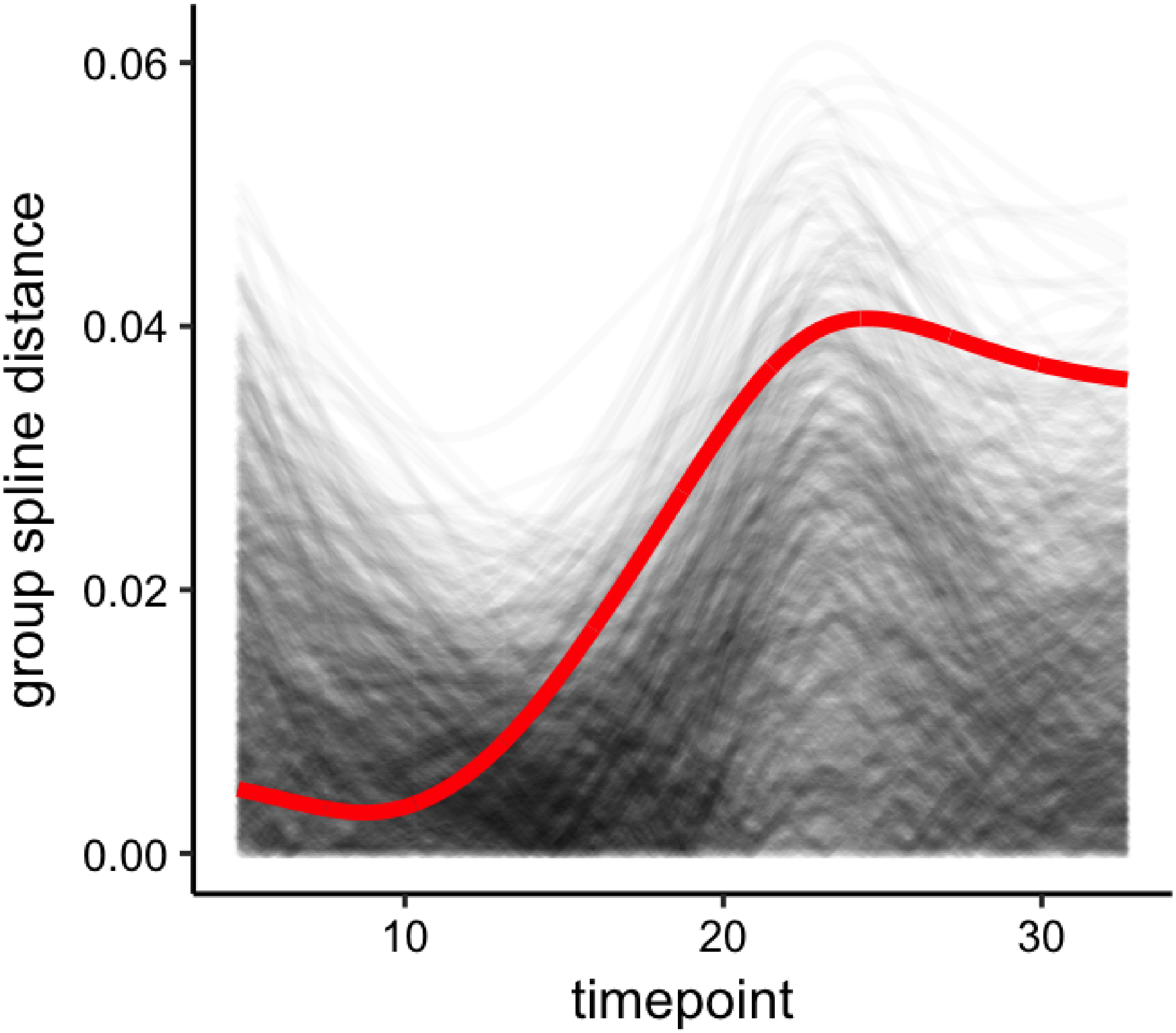

This confirms what we would expect from the previous plot, showing that our confidence of the group difference is higher once the infants pass the first year of life, and continues to become more striking through the end of the time series, where there are very few permuted lines above the observed distance.

Let's look at birth mode the same way.

```
for (f in unique(top20_family_meta$family)) {
  f__df <- top20_family_meta %>% filter(family == f)
  cat(f)
  f.result <- permuspliner(data = f__df, xvar = 'timepoint',
                           yvar = 'relative_abundance', perms = 99,
                           category = 'birth_mode', cases = 'baby_id',
                           cut_low = 6, quiet = T)
  cat('p-value =', f.result$pval)
}
```

```
## k__Bacteria.p__Actinobacteria.c__Actinobacteria.o__Bifidobacteriales.f__Bifidobacteriaceaep-value = 0
```

There are several families near significance (*Bacteroidaceae*, *Porphyromonadaceae*, *Clostridiaceae*, *Veillonellaceae*, *Pasteurellaceae*, *Enterobacteriaceae*, during the writing of this vignette) but will not astonishing after adjusting for multiple comparisons. Perhaps they are more so at a given time in the series. We can apply the

same approach using the sliding spline function.

```
birthmode_families <- list(
  'k__Bacteria.p__Bacteroidetes.c__Bacteroidia.o__Bacteroidales.f__Bacteroidaceae',
  'k__Bacteria.p__Proteobacteria.c__Gammaproteobacteria.o__Enterobacteriales.f__Enterobacteriaceae',
  'k__Bacteria.p__Firmicutes.c__Clostridia.o__Clostridiales.f__Clostridiaceae')
top20_family_meta$birth_mode <- gsub(x = top20_family_meta$birth_mode,
                                     pattern = ' ',replacement = '_', fixed = T)
slideplots <- function(xx) {
  f__df <- top20_family_meta %>% filter(family == xx)
  print(xx)
  f.result <- sliding_spliner(data = f__df, xvar = 'timepoint',
                              yvar = 'relative_abundance', category = 'birth_mode',
                              cases = 'baby_id', test_density = 3,
                              cut_low = 7, set_spar = 0.5, quiet = T)
  sliding_spliner.plot.pvals(f.result, xvar = 'timepoint')
  }
lapply(birthmode_families, FUN = slideplots)
```

```
## [1] "k__Bacteria.p__Bacteroidetes.c__Bacteroidia.o__Bacteroidales.f__Bacteroidaceae"
## [1] "k__Bacteria.p__Proteobacteria.c__Gammaproteobacteria.o__Enterobacteriales.f__Enterobacteriaceae
## [1] "k__Bacteria.p__Firmicutes.c__Clostridia.o__Clostridiales.f__Clostridiaceae"
```

```
## [[1]]
```

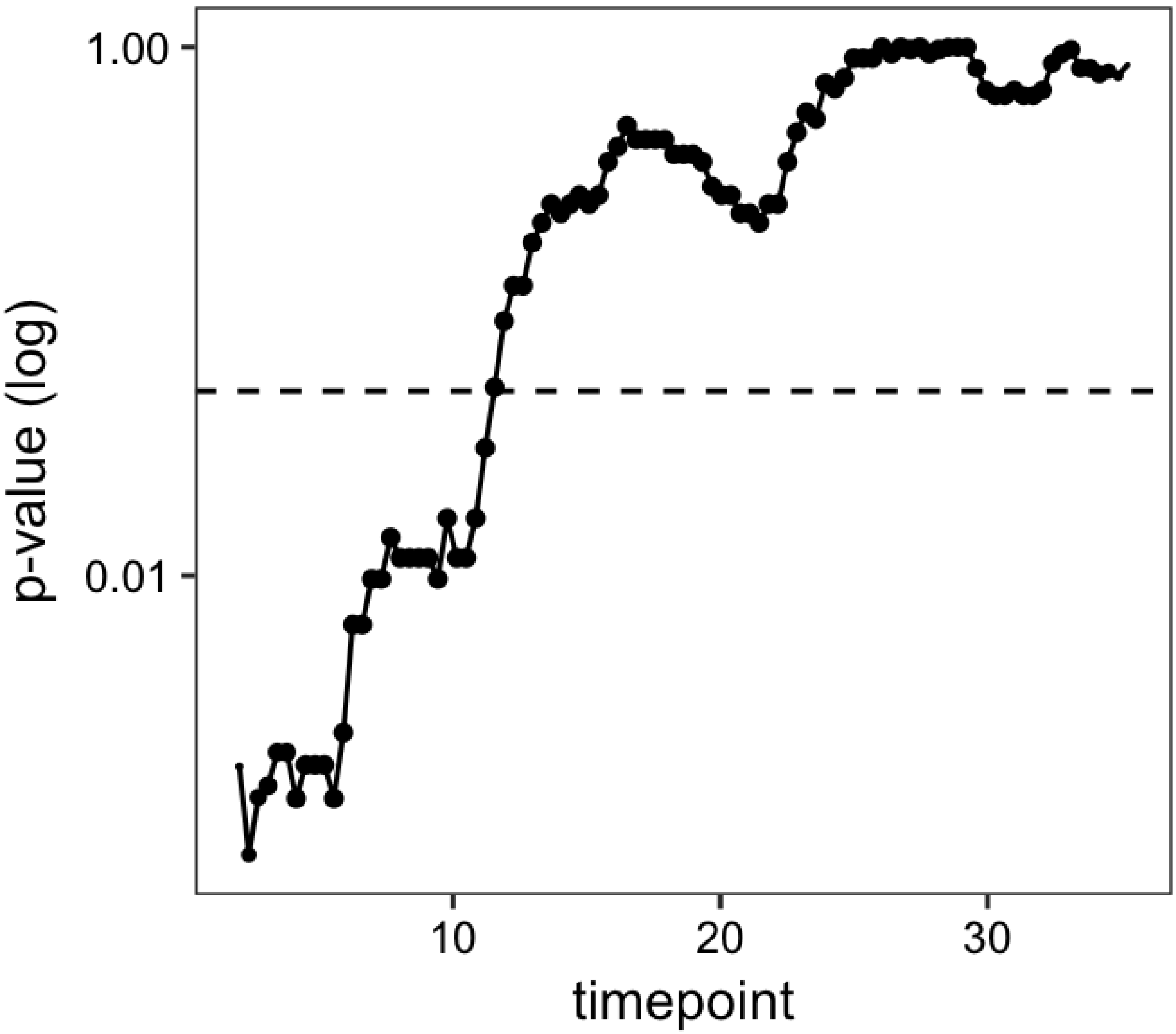


```
##
## [[2]]
```

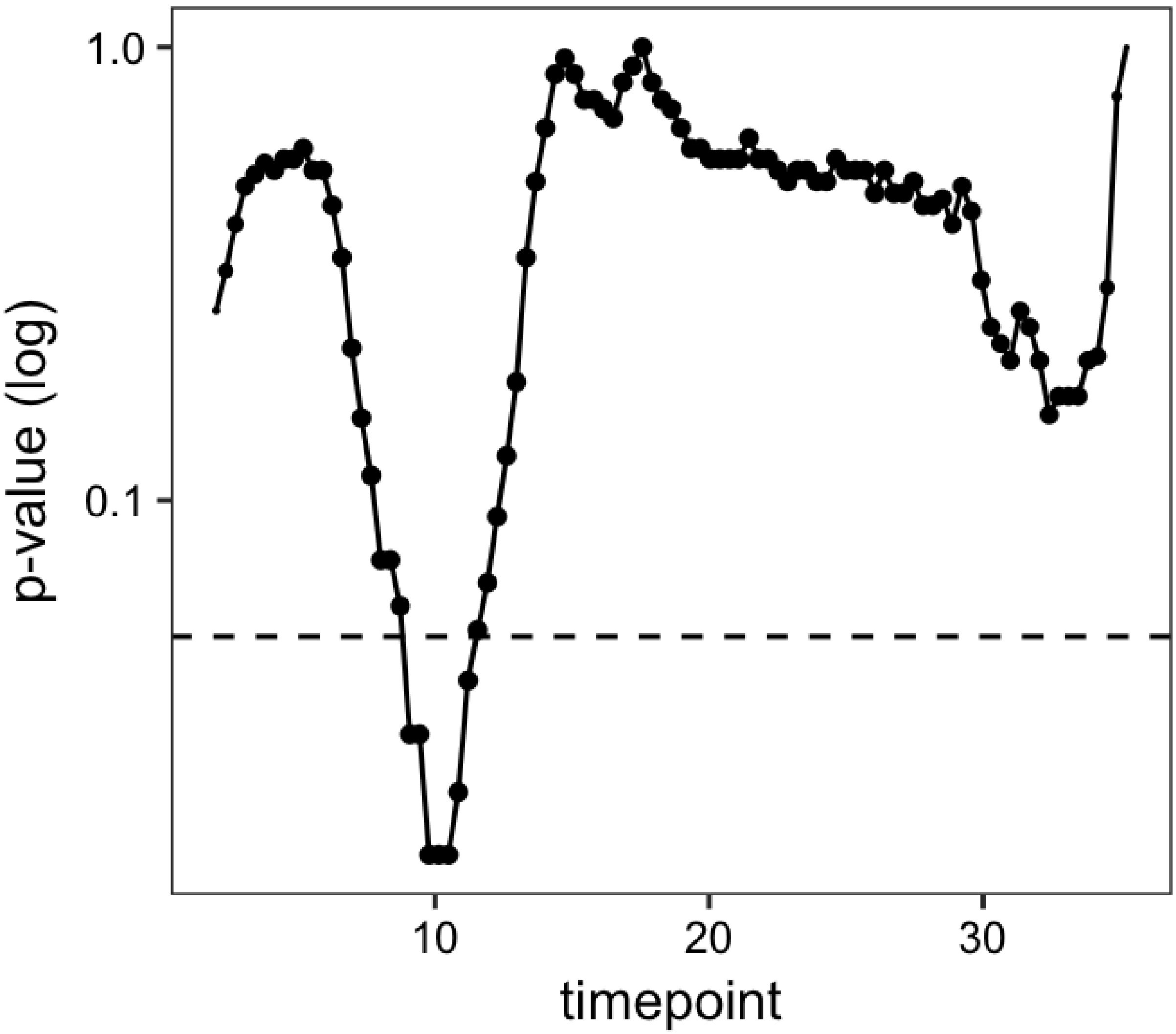


```
##
## [[3]]
```

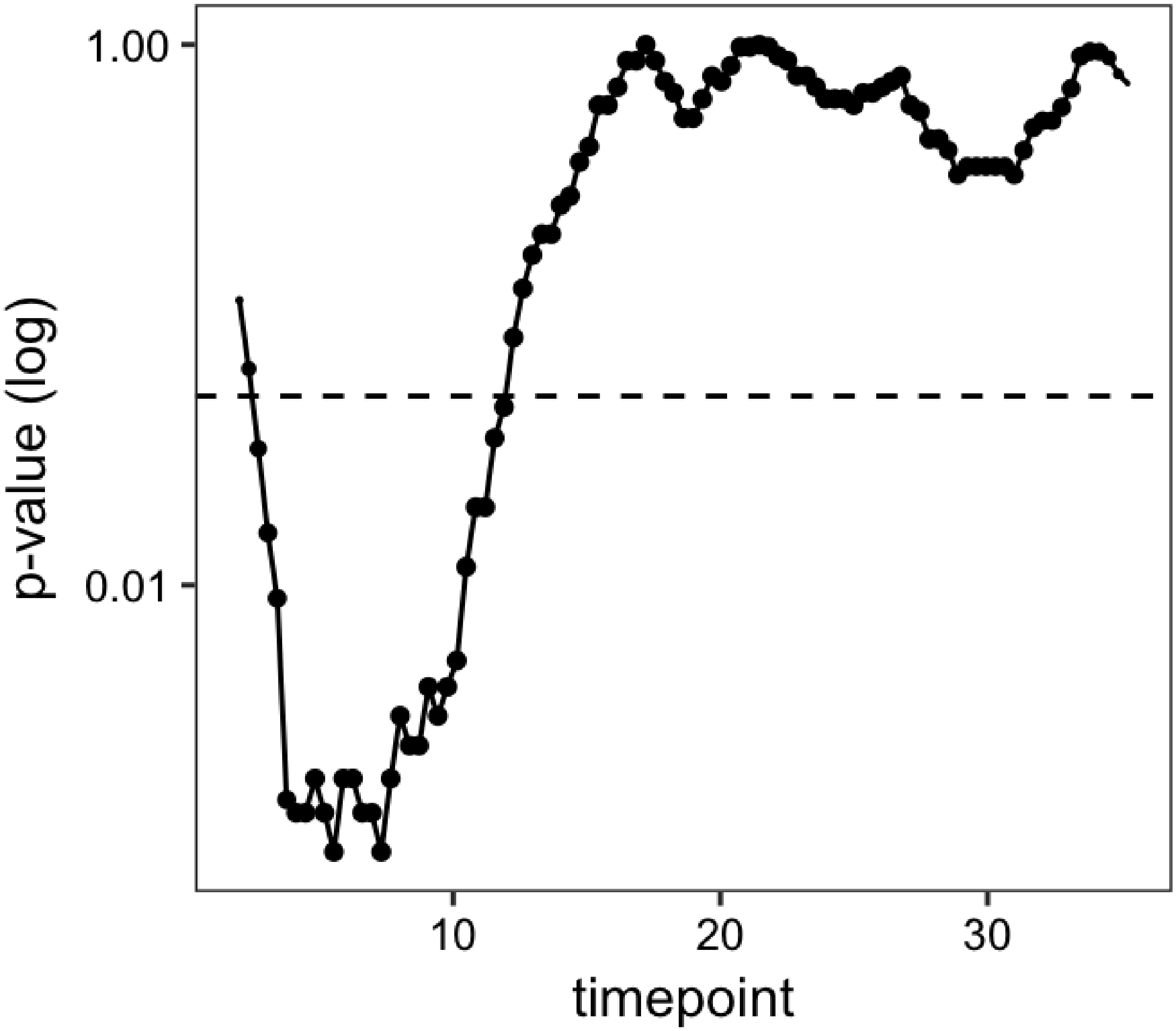

Trends in the Bacteroides genus

In the original paper, they also tested for differences in response to antibiotics at the genus level. For example, we already looked at the Family Bacteroidaceae; we can re-test their hypothesis at the Genus level, again making use of all the original time points. In the paper, this comparison is done visually by plotting individual splines over an entire page in the supplement. Using splinectomeR here allows us to summarize the groups and test whether any difference is statistically significant.

Toa test that here, first we'll prep the filtered dataframe.

```
otus_genus <- otus %>% filter(!grepl('s__', otus$OTU_ID) & grepl('g__', otus$OTU_ID))
# Run the function defined above
genus_tax_metadata <- flip_split_merge(otus_genus, metadata)
dim(genus_tax_metadata)
```

```
## [1] 1048  154
```

```
bacteroides_genus_meta <- genus_tax_metadata %>%
  gather(key = 'genus', value = 'relative_abundance',
         13:as.numeric(ncol(genus_tax_metadata))) %>%
```

```r
  filter(genus ==
'k__Bacteria.p__Bacteroidetes.c__Bacteroidia.o__Bacteroidales.f__Bacteroidaceae.g__Bacteroides')
dim(bacteroides_genus_meta)
```

```
## [1] 1048   14
```

```r
colnames(bacteroides_genus_meta)
```

```
##  [1] "SampleID"
##  [2] "baby_id"
##  [3] "birth_year"
##  [4] "sex"
##  [5] "birth_mode"
##  [6] "gestational_age"
##  [7] "months_final_exclusive_breastfeeding"
##  [8] "months_end_breastfeeding"
##  [9] "months_started_formula_feeding"
## [10] "feeding_in_hospital"
## [11] "antibiotics_y_n"
## [12] "timepoint"
## [13] "genus"
## [14] "relative_abundance"
```

Now we can run the splinectomeR test, first comparing antibiotic exposure:

```r
Bacteroides_abx_result <- permuspliner(data = bacteroides_genus_meta, xvar = 'timepoint',
                                       yvar = 'relative_abundance', cases = 'baby_id',
                                       perms = 99, category = 'antibiotics_y_n',
                                       retain_perm = T, quiet = T)
cat('p-value =', Bacteroides_abx_result$pval)
```

```
## p-value = 0.28
```

This Genus is not significantly different between antibiotic exposure groups over the time series. But, let's check birth mode, since this was something of interest in the original report:

```r
Bacteroides_birthmode_result <- permuspliner(data = bacteroides_genus_meta, xvar = 'timepoint',
                                       yvar = 'relative_abundance', cases = 'baby_id',
                                       perms = 999, category = 'birth_mode',
                                       retain_perm = F, quiet = T)
cat('p-value =', Bacteroides_birthmode_result$pval)
```

```
## p-value = 0.042
```

The test supports the conclusion that the *Bacteroides* genus is indeed significantly different in the vaginal vs. cesarean born infants over these three years. We can investigate if there is a particularly divergent region with the sliding spline test.

```r
bacteroides_slider <- sliding_spliner(data = bacteroides_genus_meta,
                                  xvar = 'timepoint', yvar = 'relative_abundance',
                                  category = 'birth_mode',
                                  cases = 'baby_id', quiet = T, test_density = 3,  # Only four C-section
                                  ints = 36, set_spar = 0.5, cut_low = 3)  # Set ints to 36 to go ~month
sliding_spliner.plot.pvals(bacteroides_slider, xvar = 'timepoint')
```

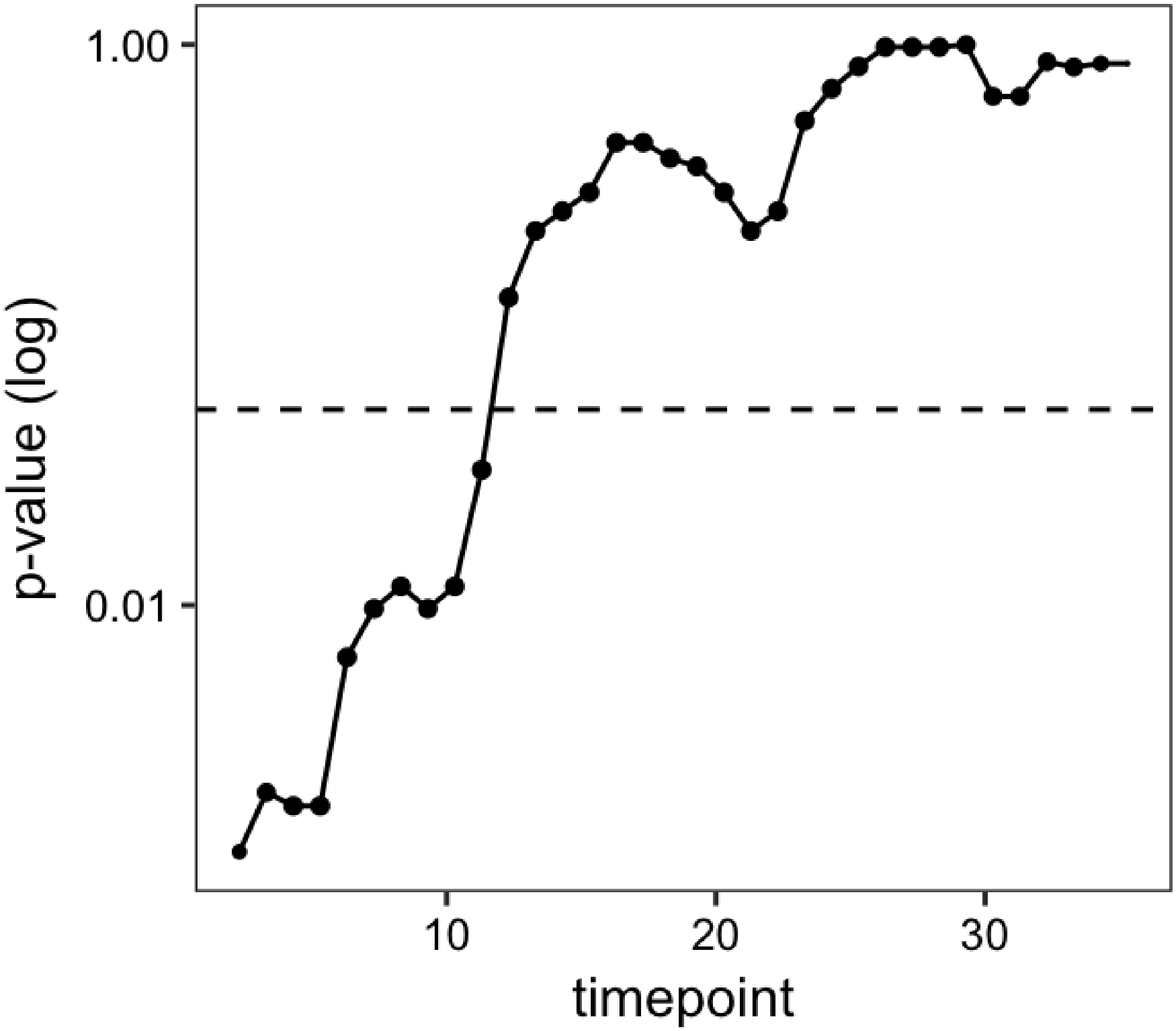

Interestingly, the biggest differences are in the first 1-2 years. But, we know from the permuspliner test that these group differences are extensive enough to be significant over the entire 36-month time series. It is worth noting that there were only four cesarean births, so the groups are weighted differently in these comparisons.

Does the ratio of Firmicutes to Bacteroidetes change over time?

One parameter that is often studied in the microbiome is the ratio of Firmicutes to Bacteroidetes (Phlya) within individuals, and the correlations between changes in the F:B ratio and risk for health conditions like obesity (Ley et al., 2006). Typically, infants have a low ratio (greater Bacteroidetes) but this is inverted in adults (Mariat et al., 2009). Let's look at this ratio across all babies.

```
# Filter and merge with mapping
otus_phyla <- otus %>% filter(!grepl('c__', otus$OTU_ID) & grepl('p__', otus$OTU_ID))
phyla_tax_metadata <- flip_split_merge(otus_phyla, metadata)

all_phyla_meta <- phyla_tax_metadata %>%
  gather(key = 'phyla', value = 'relative_abundance',
         13:as.numeric(ncol(phyla_tax_metadata)))
```

```r
# Now, unfortunately the cases need to be unique factors for the tests to work.
# So here we will just create a pseudonym for each baby's phylum abundances
all_phyla_meta$baby_id_phylum <- paste(all_phyla_meta$baby_id,
                                       all_phyla_meta$phyla, sep = '_')
head(all_phyla_meta$baby_id_phylum)
```

```
## [1] "E000823_k__Archaea.p__Euryarchaeota"
## [2] "E000823_k__Archaea.p__Euryarchaeota"
## [3] "E000823_k__Archaea.p__Euryarchaeota"
## [4] "E000823_k__Archaea.p__Euryarchaeota"
## [5] "E000823_k__Archaea.p__Euryarchaeota"
## [6] "E000823_k__Archaea.p__Euryarchaeota"
```

```r
# Now, let's use the Phyla as the category, instead of antibiotics
firm_vs_oides_result <- permuspliner(data = all_phyla_meta, xvar = 'timepoint',
                        yvar = 'relative_abundance', cases = 'baby_id_phylum',
                        perms = 999, category = 'phyla',
                        groups = c('k__Bacteria.p__Bacteroidetes','k__Bacteria.p__Firmicutes'),
                        retain_perm = T, quiet = T)
cat('p-value =', firm_vs_oides_result$pval)
```

```
## p-value = 0.001
```

But remember, these are relative abundances... So if studying two major phyla, when one goes down *in proportion*, the other expands *in proportion*. What we can do is either look at them individually (Do Bacteroidetes go up/down over time? Do Firmicutes?) or measure the ratio and then look at whether the change in the F:B ratio is non-zero over time. These questions can be answered using splinectomeR's `trendyspliner` test.

```r
phyla_tax_metadata_ratio <- phyla_tax_metadata
phyla_tax_metadata_ratio$firm_to_bact <- (phyla_tax_metadata$k__Bacteria.p__Firmicutes /
                                    phyla_tax_metadata_ratio$k__Bacteria.p__Bacteroidetes)
# Test non-zero trend here
firm_to_bact_result <- trendyspliner(data = phyla_tax_metadata_ratio,
                                     xvar = 'timepoint', yvar = 'firm_to_bact',
                                     cases = 'baby_id', mean_center = F)
```

```
##
## Testing for a non zero trend in firm_to_bact across timepoint ...
##
## Performing the trendyspline test with 999 permutations...
## Calculations completed on observed data.
##
##               Now preparing the 999 permutations - this may take some time...
## Permutations completed successfully!
##
## p-value = 0.001
```

```r
# Check the difference between birth modes
# (antibiotic use not really different, not shown)
firm_bact_birthmode_result <- permuspliner(data = phyla_tax_metadata_ratio,
                                           xvar = 'timepoint', yvar = 'firm_to_bact',
                                           cases = 'baby_id', category = 'birth_mode',
                                           quiet = T, perms = 99)
cat('Permuspliner p-value =', firm_bact_birthmode_result$pval)
```

```
## Permuspliner p-value = 0.02
```

These results tell us that the Firmicutes/Bacteroidetes ratio is indeed changing over these three years, and presents as a significantly distinct trend between C-section and vaginally born babies (with the limitation of small C-section cohort). This demonstrates how this hypothesis is quickly and straightforwardly tested with these tools. Also, the plots still show how noisy the data is – the large peaks out from the norm only result because there is considerable variability that, when shuffled in the right way, creates large differences by chance.

Let's look similarly at another ratio that is often studied in microbiome analyses: *Bacteroides* vs *Prevotella* (Genera), with the caveat that these have mostly focused on adult samples.

```
genus_tax_metadata_ratio <- genus_tax_metadata
genus_tax_metadata_ratio$prev_bact_ratio <-
  (genus_tax_metadata$k__Bacteria.p__Bacteroidetes.c__Bacteroidia.o__Bacteroidales.f__Prevotellaceae.g__
  genus_tax_metadata_ratio$k__Bacteria.p__Bacteroidetes.c__Bacteroidia.o__Bacteroidales.f__.Paraprevotel
  genus_tax_metadata$k__Bacteria.p__Bacteroidetes.c__Bacteroidia.o__Bacteroidales.f__Bacteroidaceae.g__

prev_bact_result <- trendyspliner(data = genus_tax_metadata_ratio, xvar = 'timepoint',
                                  yvar = 'prev_bact_ratio', cases = 'baby_id',
                                  mean_center = T, perms = 99, quiet = T)
cat('p-value =', prev_bact_result$pval)
```

```
## p-value = 0.02
```

```
# Check the difference between birth modes (antibiotic use not really different, not shown)
prev_bact_birthmode_result <- permuspliner(data = genus_tax_metadata_ratio,
                                           xvar = 'timepoint', yvar = 'prev_bact_ratio',
                                           cases = 'baby_id', category = 'birth_mode',
                                           perms = 99, quiet = T)
cat('p-value =', prev_bact_birthmode_result$pval)
```

```
## p-value = 0.12
```

If we look closely at the actual ratio values, they indicate that there is so little *Prevotella* present in these babies that the results are harder to interpret; *Prevotella* appears to be mostly abundant in the C-section babies, and this is what likely drives the permuspliner p value lower.

Other hypotheses we can test

Does Bifidobacterium abundance look different at early timepoints based on feeding pattern in the hospital?

```
bifidobacterium_genus_meta <- genus_tax_metadata %>%
  gather(key = 'genus', value = 'relative_abundance',
         13:as.numeric(ncol(genus_tax_metadata))) %>%
  filter(genus ==
           'k__Bacteria.p__Actinobacteria.c__Actinobacteria.o__Bifidobacteriales.f__Bifidobacteriaceae.g

head(bacteroides_genus_meta)
```

```
##       SampleID baby_id birth_year  sex       birth_mode gestational_age
## 1 E000823_25.0 E000823       2008 Male Vaginal delivery            39+4
## 2 E000823_25.7 E000823       2008 Male Vaginal delivery            39+4
## 3 E000823_29.7 E000823       2008 Male Vaginal delivery            39+4
## 4 E000823_29.6 E000823       2008 Male Vaginal delivery            39+4
## 5  E000823_5.0 E000823       2008 Male Vaginal delivery            39+4
## 6 E000823_22.6 E000823       2008 Male Vaginal delivery            39+4
```

```
##   months_final_exclusive_breastfeeding months_end_breastfeeding
## 1                                     5                    13.02
## 2                                     5                    13.02
## 3                                     5                    13.02
## 4                                     5                    13.02
## 5                                     5                    13.02
## 6                                     5                    13.02
##   months_started_formula_feeding feeding_in_hospital antibiotics_y_n
## 1                             NA      OnlyBreastmilk               N
## 2                             NA      OnlyBreastmilk               N
## 3                             NA      OnlyBreastmilk               N
## 4                             NA      OnlyBreastmilk               N
## 5                             NA      OnlyBreastmilk               N
## 6                             NA      OnlyBreastmilk               N
##   timepoint
## 1      25.0
## 2      25.7
## 3      29.7
## 4      29.6
## 5       5.0
## 6      22.6
##                                                                                           genus
## 1 k__Bacteria.p__Bacteroidetes.c__Bacteroidia.o__Bacteroidales.f__Bacteroidaceae.g__Bacteroides
## 2 k__Bacteria.p__Bacteroidetes.c__Bacteroidia.o__Bacteroidales.f__Bacteroidaceae.g__Bacteroides
## 3 k__Bacteria.p__Bacteroidetes.c__Bacteroidia.o__Bacteroidales.f__Bacteroidaceae.g__Bacteroides
## 4 k__Bacteria.p__Bacteroidetes.c__Bacteroidia.o__Bacteroidales.f__Bacteroidaceae.g__Bacteroides
## 5 k__Bacteria.p__Bacteroidetes.c__Bacteroidia.o__Bacteroidales.f__Bacteroidaceae.g__Bacteroides
## 6 k__Bacteria.p__Bacteroidetes.c__Bacteroidia.o__Bacteroidales.f__Bacteroidaceae.g__Bacteroides
##   relative_abundance
## 1           0.328744
## 2           0.482087
## 3           0.654723
## 4           0.479338
## 5           0.499211
## 6           0.585621
```

```
bifido_slider <- sliding_spliner(data = bifidobacterium_genus_meta,
                                 xvar = 'timepoint', yvar = 'relative_abundance',
                                 category = 'feeding_in_hospital', quiet = T,
                                 cases = 'baby_id', test_density = 10, cut_low = 7,
                                 set_spar = 0.5, groups = c('OnlyBreastmilk','mixed'))
sliding_spliner.plot.pvals(bifido_slider, xvar = 'timepoint')
```

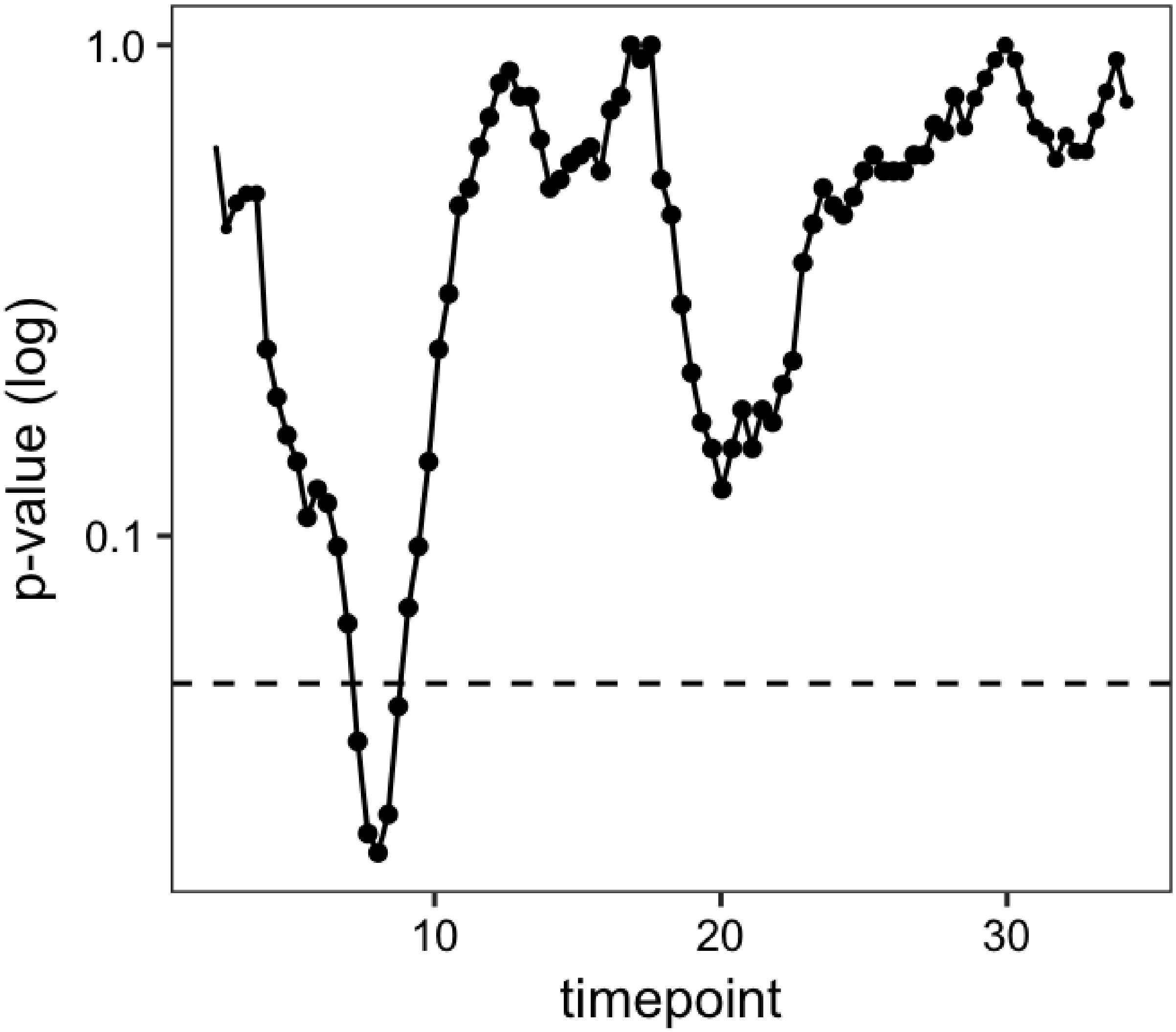

This plot there's something about the period where many of the babies are beginning to wean that is different between these groups. The average age when weaning can be reported easily:

```
mean(as.numeric(metadata$months_end_breastfeeding))
```

```
## [1] 9.724211
```

Are there differences in Clostridium groups IV and XIVa between the antibiotic +/- groups?

Clostridium groups IV and XIVa are of interest in microbiome studies because they are butyrate producers, butyrate being one of the primary short chain fatty acids that may be mechanistically involved in some of the health consequences associated with microbiome composition. I'm including the *Faecalibacterium* genus as a member of cluster IV, though it may not be universally included, it appears.

In the paper, they use the relative abundance at a single timepoint to test the difference (Fig. S8). With splinectomeR, we can use all of the data across the entire time series to test whether antibiotic exposure status is associated with different levels of these butyrate producers.

```r
# Define the Genera in these Clostridium clusters (that are present in this dataset)
clostrid_groups <- c(
  'k__Bacteria|p__Firmicutes|c__Clostridia|o__Clostridiales|f__Clostridiaceae|g__Clostridium',
  'k__Bacteria|p__Firmicutes|c__Clostridia|o__Clostridiales|f__Lachnospiraceae|g__Coprococcus',
  'k__Bacteria|p__Firmicutes|c__Clostridia|o__Clostridiales|f__Lachnospiraceae|g__Dorea',
  'k__Bacteria|p__Firmicutes|c__Clostridia|o__Clostridiales|f__Lachnospiraceae|g__Lachnospira',
  'k__Bacteria|p__Firmicutes|c__Clostridia|o__Clostridiales|f__Lachnospiraceae|g__Roseburia',
  'k__Bacteria|p__Firmicutes|c__Clostridia|o__Clostridiales|f__Lachnospiraceae|g__[Ruminococcus]',
  'k__Bacteria|p__Firmicutes|c__Clostridia|o__Clostridiales|f__Ruminococcaceae|g__Ruminococcus',
  'k__Bacteria|p__Firmicutes|c__Clostridia|o__Clostridiales|f__Ruminococcaceae|g__Faecalibacterium')
# Filter and merge
otus_clostrid <- otus %>% filter(OTU_ID %in% clostrid_groups)
clostrid_tax_metadata <- flip_split_merge(otus_clostrid, metadata)
clostrid_tax_metadata$clostridiales_abundance <-
  rowSums(x = clostrid_tax_metadata[, 13:ncol(clostrid_tax_metadata)])

colnames(clostrid_tax_metadata)
```

```
##  [1] "SampleID"
##  [2] "baby_id"
##  [3] "birth_year"
##  [4] "sex"
##  [5] "birth_mode"
##  [6] "gestational_age"
##  [7] "months_final_exclusive_breastfeeding"
##  [8] "months_end_breastfeeding"
##  [9] "months_started_formula_feeding"
## [10] "feeding_in_hospital"
## [11] "antibiotics_y_n"
## [12] "timepoint"
## [13] "k__Bacteria.p__Firmicutes.c__Clostridia.o__Clostridiales.f__Clostridiaceae.g__Clostridium"
## [14] "k__Bacteria.p__Firmicutes.c__Clostridia.o__Clostridiales.f__Lachnospiraceae.g__Coprococcus"
## [15] "k__Bacteria.p__Firmicutes.c__Clostridia.o__Clostridiales.f__Lachnospiraceae.g__Dorea"
## [16] "k__Bacteria.p__Firmicutes.c__Clostridia.o__Clostridiales.f__Lachnospiraceae.g__Lachnospira"
## [17] "k__Bacteria.p__Firmicutes.c__Clostridia.o__Clostridiales.f__Lachnospiraceae.g__Roseburia"
## [18] "k__Bacteria.p__Firmicutes.c__Clostridia.o__Clostridiales.f__Lachnospiraceae.g__.Ruminococcus."
## [19] "k__Bacteria.p__Firmicutes.c__Clostridia.o__Clostridiales.f__Ruminococcaceae.g__Faecalibacteriu
## [20] "k__Bacteria.p__Firmicutes.c__Clostridia.o__Clostridiales.f__Ruminococcaceae.g__Ruminococcus"
## [21] "clostridiales_abundance"
```

```r
clostrid_result <- permuspliner(data = clostrid_tax_metadata,
                                xvar = 'timepoint', yvar = 'clostridiales_abundance',
                                category = 'antibiotics_y_n', quiet = T,
                                cases = 'baby_id', retain_perm = T)
cat('p-value =', clostrid_result$pval)
```

```
## p-value = 0.003
```

Noteably, this is a *stronger* result than when comparing relative abundance at 36 months, and provides even more support for their conclusions. We can also look at the plot to see where the differences mostly lie temporally.

```r
permuspliner.plot.permsplines(clostrid_result, xvar = 'timepoint', yvar = 'clostridiales_abundance')
```

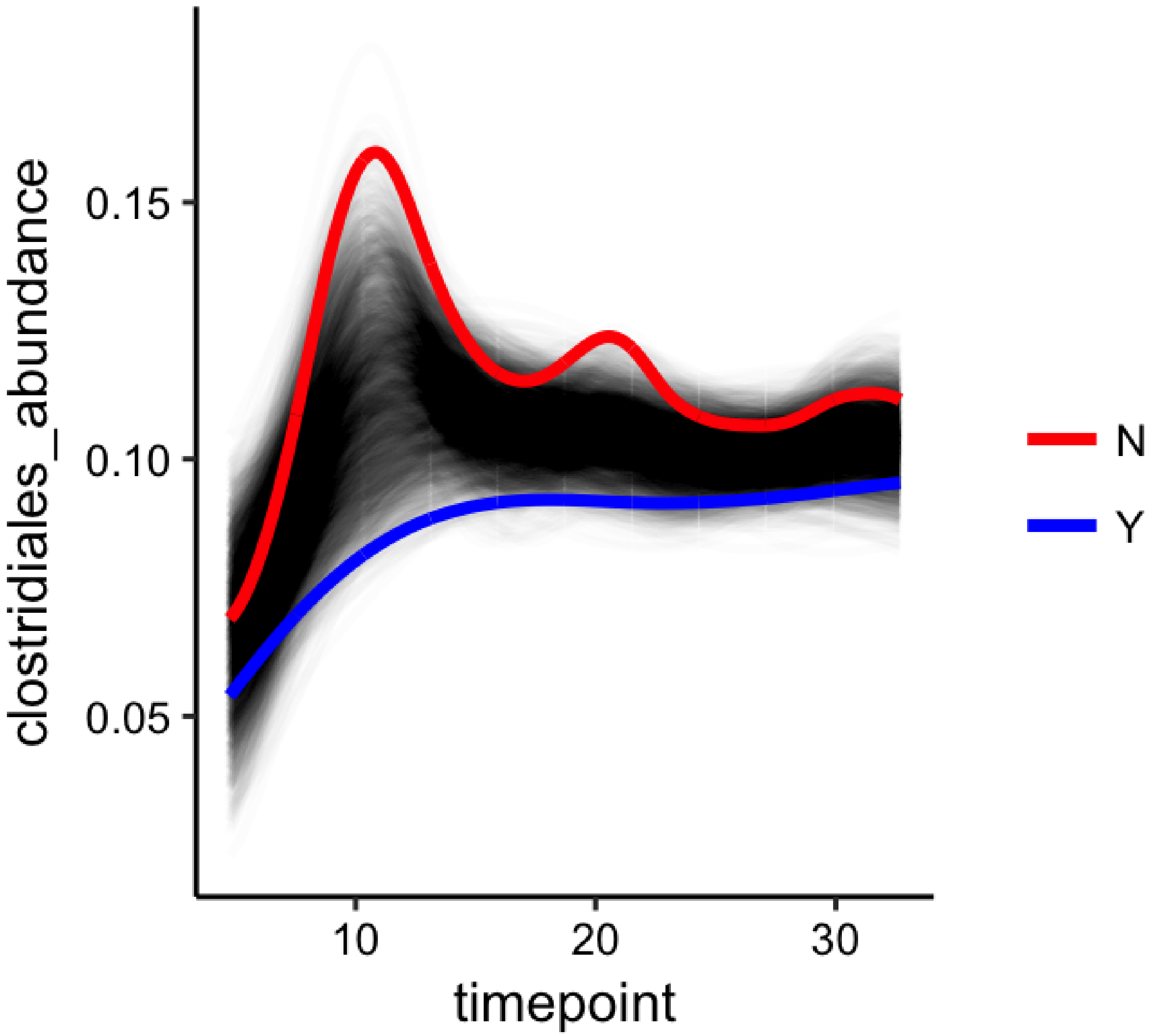


```
permuspliner.plot.permdistance(clostrid_result, xlabel = 'timepoint')
```

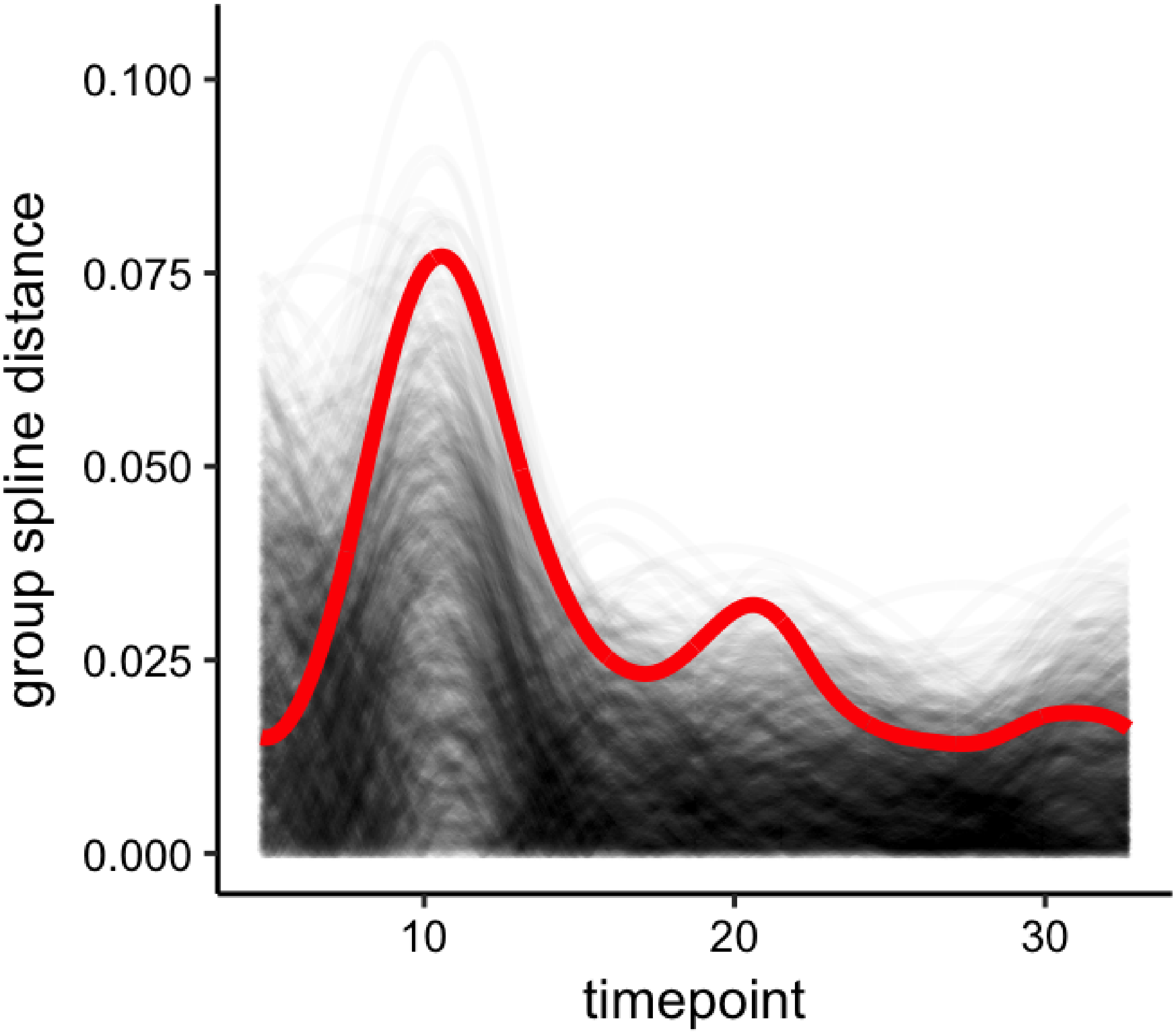

Interesting - they nearly converge where the authors were testing the difference (36 months). Looking across the time series gives a more complete answer, and in this case it is statistically more powerful as well. Side note: if you remove *Faecalibacterium*, the difference even slightly stronger overall but at 36 months they are much more similar; therefore, without testing across the full time course, as enabled by splinectomeR, you would not detect a difference with that timepoint.

Does alpha diversity differ, or change differently, over time?

First, calculate the Shannon diversity (using the `vegan` package) from the OTU table, and add the metadata. We don't have the raw counts, so we will just filter to a Genus table and calculate the Shannon diversity from that.

```
otus_genus <- otus %>% filter(!grepl('s__', otus$OTU_ID) & grepl('g__', otus$OTU_ID))
alphadiv <- as.data.frame(diversity(t(otus_genus[,2:ncol(otus_genus)]), 'shannon'))
alphadiv <- tibble::rownames_to_column(alphadiv, var = 'sampleID')
colnames(alphadiv)[2] <- 'shannon'
alphadiv <- alphadiv %>% separate(sampleID, c('baby_id', 'timepoint'), sep = '_')
alphadiv$timepoint <- as.numeric(alphadiv$timepoint)
alphadiv$shannon <- as.numeric(alphadiv$shannon)
```

```r
alphadiv_meta <- merge(metadata, alphadiv, by = 'baby_id')
```

Is alpha diversity different over time between antibiotic status or birth mode?

```r
alpha_permresult_abx <- permuspliner(data = alphadiv_meta, xvar = 'timepoint',
                                    yvar = 'shannon', category = 'antibiotics_y_n',
                                    cases = 'baby_id', perms = 99, retain_perm = T,
                                    quiet = T)

alpha_permresult_mode <- permuspliner(data = alphadiv_meta, xvar = 'timepoint',
                                    yvar = 'shannon', category = 'birth_mode',
                                    cases = 'baby_id', perms = 99, retain_perm = T,
                                    quiet = T)

p_abx <- permuspliner.plot.permsplines(data = alpha_permresult_abx,
                                      xvar = 'timepoint', yvar = 'shannon')
p_mode <- permuspliner.plot.permsplines(data = alpha_permresult_mode,
                                      xvar = 'timepoint', yvar = 'shannon')
p_abx
```

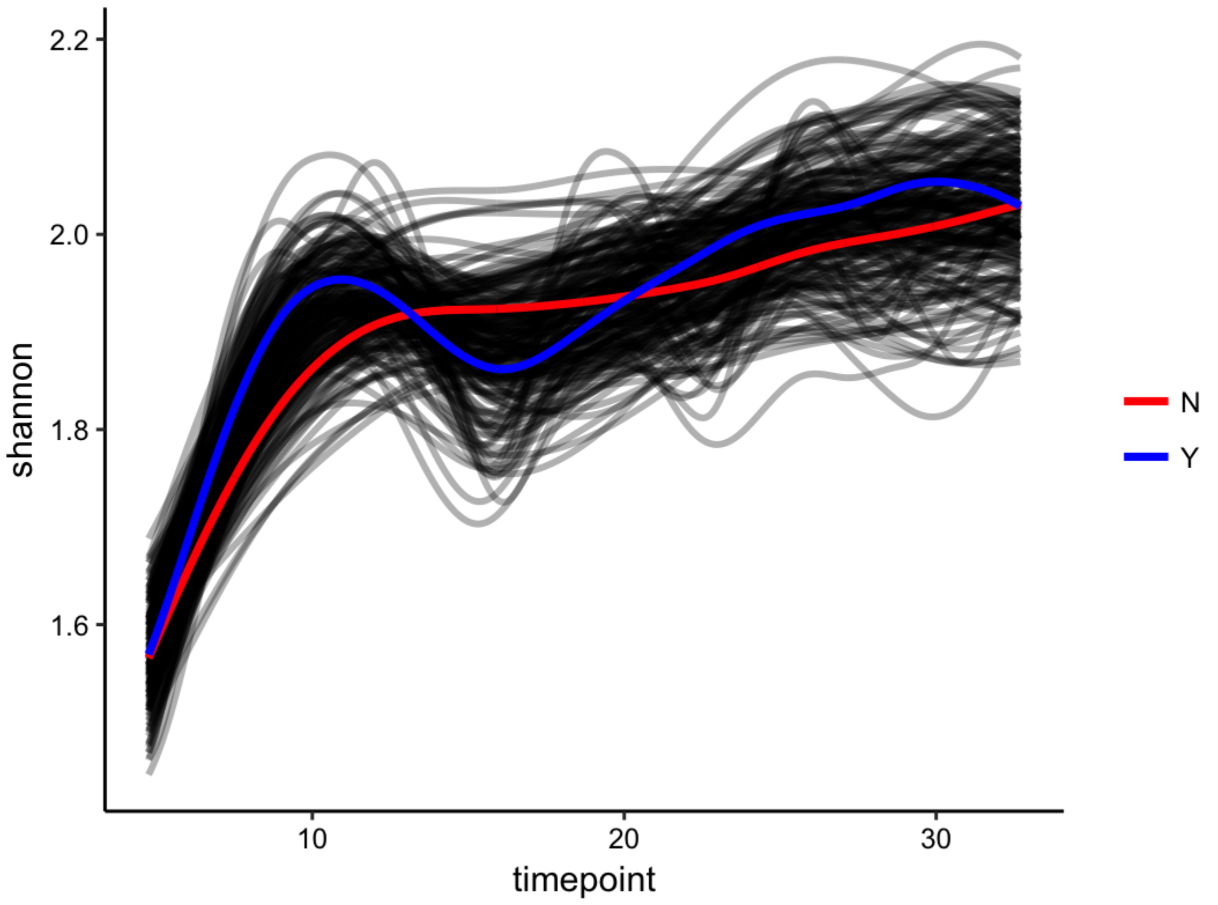


```r
p_mode
```

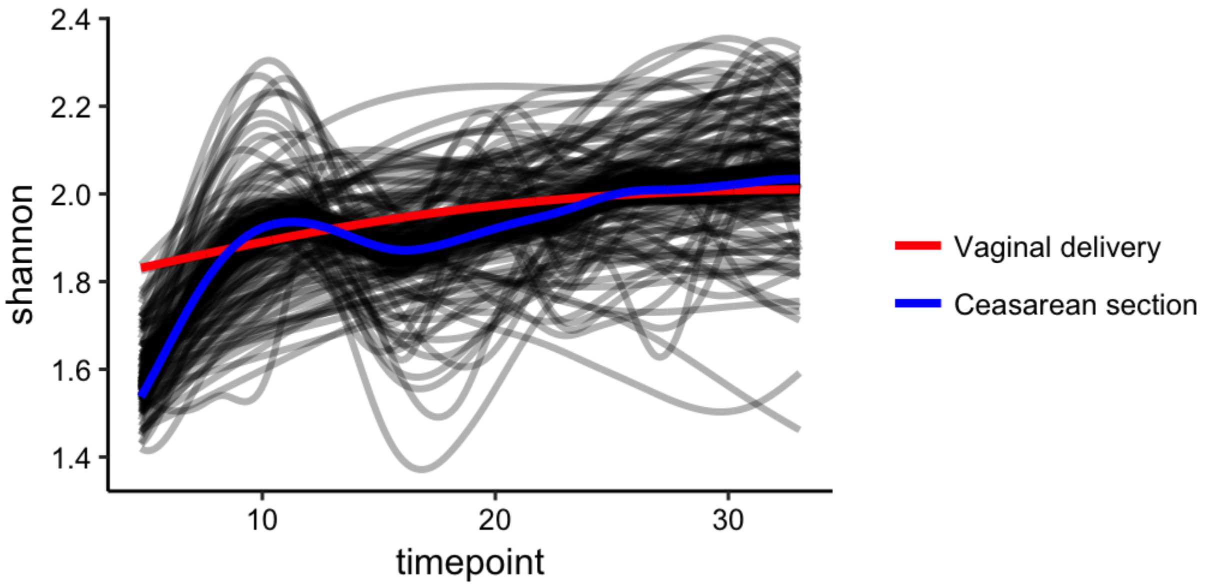

It looks like alpha diversity is increasing in these babies, regardless their antibiotic exposure or birth mode, and that increase is fairly similar in rate and shape between these environmental factors.
We can also look more finely at the differences at each interval of time.

```
alpha_slide <- sliding_spliner(data = alphadiv_meta, xvar = 'timepoint',
                               yvar = 'shannon', category = 'birth_mode',
                               cases = 'baby_id', set_spar = 0.5, quiet = T)
sliding_spliner.plot.pvals(alpha_slide, xvar = 'timepoint')
```

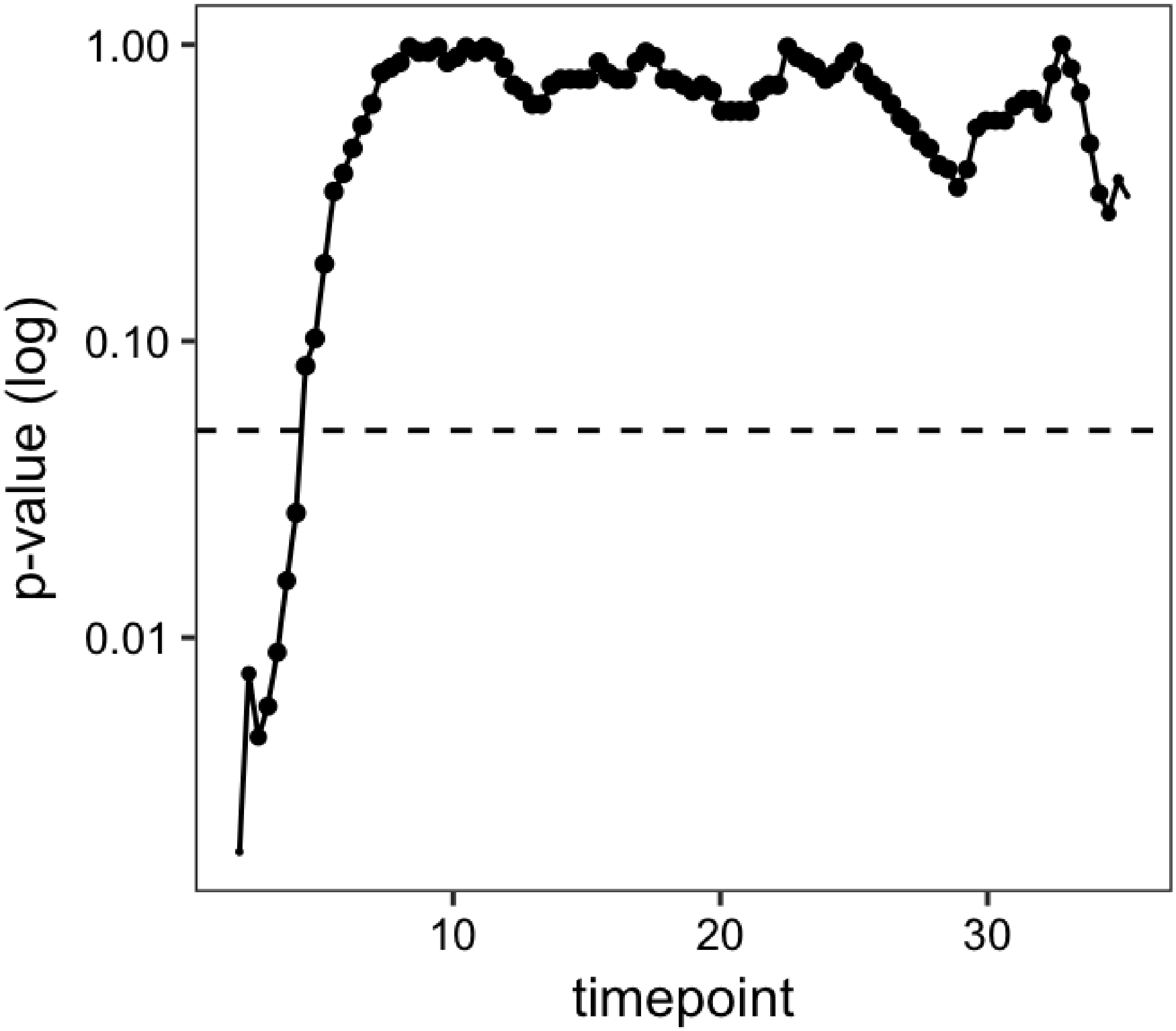

Another hypothesis surrounding babies in the first years of life is that their overall diversity should be increasing as they get older. We can confirm this with our tests, by asking whether alpha diversity is non-linear over time. We can also test this within the groups of antibiotic exposed and non-exposed babies.

```
alpha_trendresult_all <- trendyspliner(data = alphadiv_meta, xvar = 'timepoint',
                                       yvar = 'shannon', cases = 'baby_id',
                                       perms = 99, quiet = T)
cat('p-value =', alpha_trendresult_all$pval)
```

```
## p-value = 0.01
```

```
# And to visualize the trend in the context of the permuted data
trendyspliner.plot.perms(alpha_trendresult_all, xlabel = 'time', ylabel = 'Shannon diversity')
```

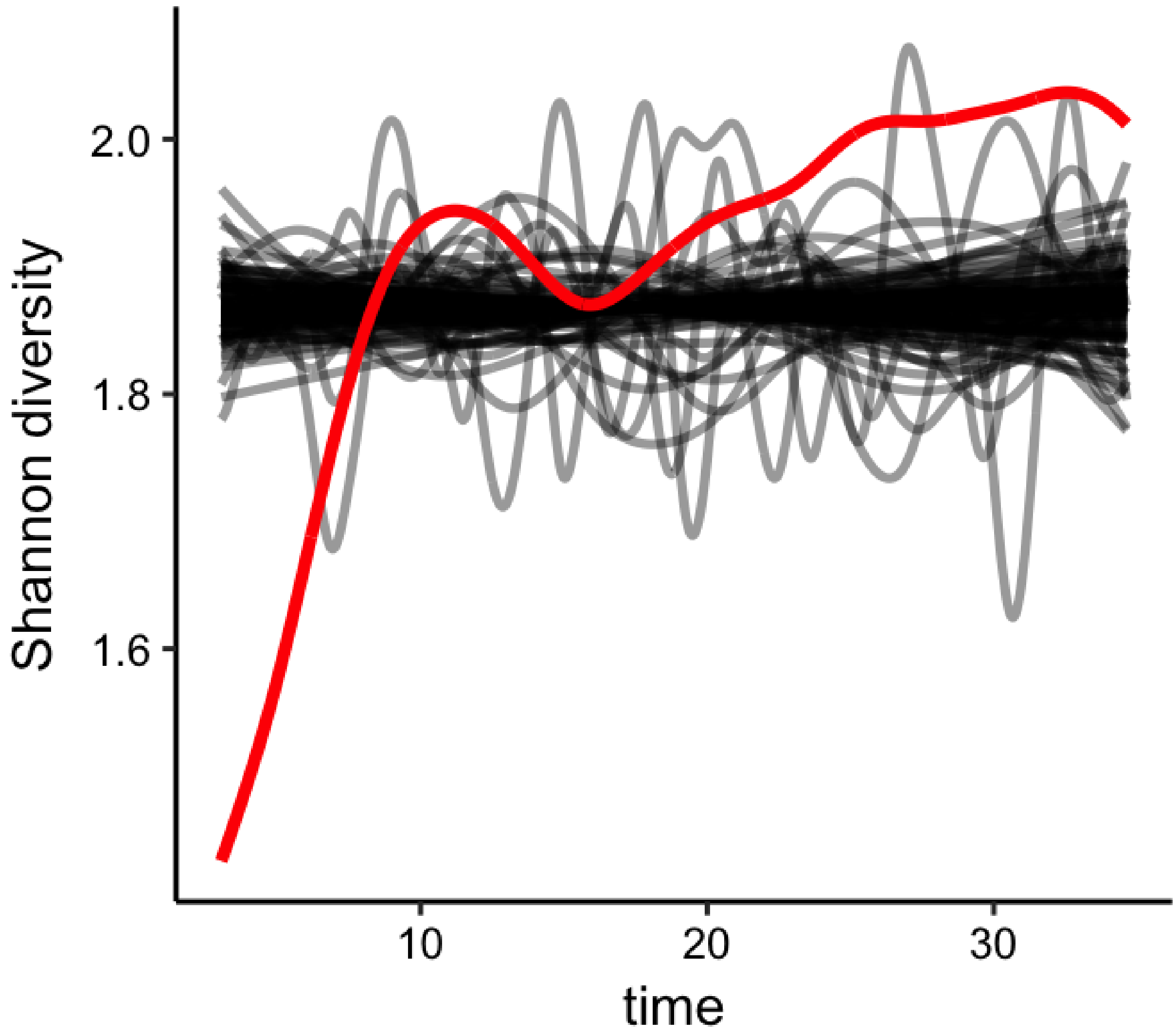


```
# Now in antibiotic exposed infants
alpha_trendresult_abx <- trendyspliner(data = alphadiv_meta, xvar = 'timepoint',
                                       yvar = 'shannon', cases = 'baby_id', perms = 99,
                                       category = 'antibiotics_y_n',
                                       group = 'Y', quiet = T)
cat('p-value =', alpha_trendresult_abx$pval)
```

```
## p-value = 0.01
```

```
# And in infants not exposed to antibiotics
alpha_trendresult_noabx <- trendyspliner(data = alphadiv_meta, xvar = 'timepoint',
                                         yvar = 'shannon', cases = 'baby_id',
                                         perms = 99, quiet = T,
                                         category = 'antibiotics_y_n', group = 'N')
cat('p-value =', alpha_trendresult_noabx$pval)
```

```
## p-value = 0.01
```

Alpha diversity increases significantly in both groups, but the trends are not significantly different over time between antibiotics (from the previous tests).

Conclusions

Yassour et al. present a monumental dataset with dense longitudinal data that details the human gut microbiome's complex dynamics over the first three years of life. They also have recorded data on several variables which the microbiome patterns can be investigated, including antibiotic exposure, delivery mode, and feeding patterns.

A remaining challenge in analyses like these is a lack of clear, simple methods to directly compare data across multiple individuals and many time points–in particular when those time points are not in sync–without averaging or adding across multiple points, or subsetting and dropping many of the data points. In both cases, the longitudinal density is not being leveraged to its full possible extent.

In this vignette, we've shown how these `splinectomeR` tools can quickly assess statistical significance by summarizing group data with splines and generating randomly permuted distributions to evaluate the probability that the observed magnitude of difference is due to chance. By employing the three test functions, we can explore overall changes in abundances or metrics, and compare longitudinal trends between two categorical groups.

The approaches here provide statistical support for proposals the authors have drawn, and allow us to test supplemental hypotheses. Importantly, we are able to perform these tests in a way that takes full advantage of the longitudinal data and maintains the individual observations.
